# Cell-specific NFIA upregulation promotes epileptogenesis by TRPV4-mediated astrocyte reactivity

**DOI:** 10.1186/s12974-023-02909-4

**Published:** 2023-10-25

**Authors:** Shuo Kong, Tao-xiang Chen, Xiang-lei Jia, Xue-lei Cheng, Meng-liu Zeng, Jing-yi Liang, Xiao-hua He, Jun Yin, Song Han, Wan-hong Liu, Yuan-teng Fan, Ting Zhou, Yu-min Liu, Bi-wen Peng

**Affiliations:** 1 Department of Physiology, Hubei Provincial Key Laboratory of Developmentally Originated Disease, School of Basic Medical Sciences, Wuhan University, Wuhan, China; 2 Department of Pathophysiology, School of Basic Medical Sciences, Wuhan University, Wuhan, China; 3 Department of Immunology, School of Basic Medical Sciences, Wuhan University, Wuhan, China; 4 Department of Neurology, Zhongnan Hospital, Wuhan University, Donghu Road 169#, Wuhan, 430071 China; 5 Department of Neurology, People’s Hospital of Dongxihu District, Wuhan, 430040 Hubei China

**Keywords:** NFIA, TRPV4, Epilepsy, Reactive astrocyte, Neuroinflammation

## Abstract

**Background:**

The astrocytes in the central nervous system (CNS) exhibit morphological and functional diversity in brain region-specific pattern. Functional alterations of reactive astrocytes are commonly present in human temporal lobe epilepsy (TLE) cases, meanwhile the neuroinflammation mediated by reactive astrocytes may advance the development of hippocampal epilepsy in animal models. Nuclear factor I-A (NFIA) may regulate astrocyte diversity in the adult brain. However, whether NFIA endows the astrocytes with regional specificity to be involved in epileptogenesis remains elusive.

**Methods:**

Here, we utilize an interference RNA targeting NFIA to explore the characteristics of NFIA expression and its role in astrocyte reactivity in a 4-aminopyridine (4-AP)-induced seizure model in vivo and in vitro. Combined with the employment of a HA-tagged plasmid overexpressing NFIA, we further investigate the precise mechanisms how NIFA facilitates epileptogenesis.

**Results:**

4-AP-induced NFIA upregulation in hippocampal region is astrocyte-specific, and primarily promotes detrimental actions of reactive astrocyte. In line with this phenomenon, both NFIA and vanilloid transient receptor potential 4 (TRPV4) are upregulated in hippocampal astrocytes in human samples from the TLE surgical patients and mouse samples with intraperitoneal 4-AP. NFIA directly regulates mouse astrocytic TRPV4 expression while the quantity and the functional activity of TRPV4 are required for 4-AP-induced astrocyte reactivity and release of proinflammatory cytokines in the charge of NFIA upregulation. NFIA deficiency efficiently inhibits 4-AP-induced TRPV4 upregulation, weakens astrocytic calcium activity and specific astrocyte reactivity, thereby mitigating aberrant neuronal discharges and neuronal damage, and suppressing epileptic seizure.

**Conclusions:**

Our results uncover the critical role of NFIA in astrocyte reactivity and illustrate how epileptogenic brain injury initiates cell-specific signaling pathway to dictate the astrocyte responses.

**Supplementary Information:**

The online version contains supplementary material available at 10.1186/s12974-023-02909-4.

## Background

Epileptic seizures are widely believed to be a result of the excitation–inhibition imbalance of neuronal activities in the central nervous system (CNS). Although hippocampal lesions are thought to be an ascertained cause for temporal lobe epilepsy (TLE), the most common form of adult refractory epilepsy, there is no clinically validated antiepileptogenic therapy for prevention of epileptic recurrence due to many different neurobiological processes including neuroinflammation induced by the epileptogenic insult [1–3]. The fundamental processes that promote epileptogenesis are still to be established.

Astrocytes are crucial stabilizers of the normal neuronal excitability based on their buffering extracellular potassium ions and timely clearing synaptic neurotransmitters as well as releasing gliotransmitters, and they are also critical regulators in the CNS immune microenvironment in some brain diseases such as glioblastoma, multiple sclerosis as well as epilepsy [4–6]. Reactive astrocytes after neurological injuries frequently lose their original function of stabilizing neuronal excitability, and exhibit morphological and functional alterations with different gene expression profiles [7]. Distinct transcriptional patterns implicate optional genetic programs and different signaling mechanisms to establish astrocyte subpopulations [8–10], thus endowing the astrocytes with distinct functions and producing different effects on neocortical and hippocampal epileptogenesis [11–14].

NFIA is regarded as a key transcriptional node in regulating astrocyte responses [16], and also a primary contributor for astrocyte to oversee hippocampal circuit activities in a region-specific transcriptional pattern [17]. Knocking down NFIA can impair hippocampal astrocytic calcium activity possibly by reducing its target channel protein-vanilloid transient receptor potential 4 (TRPV4) [17]. Our recent study confirmed that TRPV4 and glial fibrillary acidic protein (GFAP) are synchronously upregulated in human hippocampal tissues from TLE patients and a 4-aminopyridine (4-AP)-induced epilepsy mice, and also explored the functional activity of TRPV4 promotes the reactivity of astrocytes [18].

Given the role of NFIA in astrocytic reaction and calcium activity, and the ability of TRPV4 channel permeable to calcium ions, we hypothesized that NFIA, as a transcription factor, may regulate the expression of TRPV4 to alter astrocyte reactivity, thus facilitating 4-AP-induced seizures in mice. We found that 4-AP-induced NFIA in an astrocyte-specific pattern transcriptionally upregulates TRPV4 expression and aggravates astrocyte-mediated neuroinflammation via the functional activity of TRPV4.

## Methods

### Animals

8-week-old male C57BL/6 wild-type mice (weighing 20 ± 3 g) were purchased from the Hubei Province Center for Animal Experiments. All animal experiments were performed according to the Institutional Animal Care and Use Committee of Wuhan University Medical School and the National Institutes of Health Guide for the Care and Use of Laboratory Animals (NIH Publications No. 8023, revised 1978). Mice were grouped randomly and maintained at the ambient temperature of 25 ± 1 °C and the relative humidity of 60~80% with a 12-h light–dark cycle; food and water were available ad libitum in the animal biosafety level III laboratory (ABSL-III) of Wuhan University.

### Human brain specimens

Experimental procedure involving humans was performed according to the World Medical Association Declaration of Helsinki (2000) and was approved by the Medical Ethics Committee of Zhongnan Hospital of Wuhan University. All human brain specimens were collected either from the TLE patients who had surgery (5, male, 25~58 years) at the Department of Neurosurgery of Zhongnan Hospital (Wuhan University, Wuhan, China) or from the deceased who had no history of seizures or other neurological diseases and postmortem within 24 h of death (6, male, 26~57 years) at Zhongnan Hospital.

### Hippocampal stereotactic injection and electrode implantation

Freely moving mice were subcutaneously injected with ketoprofen (5 mg/kg, MCE, China, # RP-19583), and one hour later they were anesthetized by isoflurane inhalation (5% for induction and 2% for maintenance, RWD, China, #R510-22-10), and mounted in a stereotaxic apparatus for performing the delivery of interference RNA (siRNA) or recombinant adeno-associated virus (rAAV) and electroencephalogram (EEG) recording electrode implantation. The specific siRNA targeting NFIA (si.NFIA) and non-specific siRNA (si.NC) as a control (GenePharma Company, China) were used for universal interference of NFIA while rAAV-GfaABC1D-EGFP-5′miR-30a-shRNA(mNFIA)-3′miR-30a-WPREs (rAAV-GfaABC1D-sh.NFIA) and rAAV-GfaABC1D-EGFP-5′miR-30a-shRNA(scramble)-3′miR-30a-WPREs (rAAV-GfaABC1D-sh.NC) were used for astrocyte-specific interference of NFIA (BrainCase Company, China). The si.NFIA and rAAV-GfaABC1D-sh.NFIA share the same sequences: GGCCAAGUUACGGAAAGAUTT (forward primer) and AUCUUUCCGUAACUUGGCCTT (reverse prime). 6 μl siRNA or 1 μl rAAV with the concentration of 5 × 10^12^ genome copies per ml was injected unilaterally into hippocampal CA1 region (1 mm posterior to bregma, 1.7 mm lateral lobe, 1.85 mm deep from the pial surface). 10 min later, two twisted silver steel electrodes were implanted bilaterally into the same region and fixed by soft denture resin.

### Mouse model of acute seizure

Intraperitoneal injection of 4-AP (5.6 mg/kg, Sigma-Aldrich, USA, #504-24-5) was used to establish in vivo mouse model of acute seizure. Behavioral evaluation of seizure severity was mainly based on a modified Racine scale: (I) arrest and rigid posture; (II) head nodding; (III) unilateral forelimb clonus; (IV) bilateral forelimb clonus; (V) forelimb and hindlimb clonus with falling; (VI) tonic–clonic seizure with running and jumping; and (VII) death [19, 20].The EEG signals were digitized and analyzed with Lab Chart software (AD Instruments, Bella Vista, New South Wales, Australia). Addition of 4-AP (5 mM) to the culture medium for primary cells was used to establish in vitro mouse model of acute seizure.

### Whole-cell patch recording

The hippocampal slices were prepared and the electrical activity of pyramidal neurons in CA1 region was detected by whole-cell current clamp with a patch clamp device of AXON 700B and Digidata 1550 (Axon, Molecular Device) at 22 ± 2 °C at Medical Research Center for Structural Biology of Wuhan University. The bath solution (in mM) consisted of 140 NaCl, 5 KCl, 2 MgCl_2_, 2 CaCl_2_, 10 HEPES, and 10 glucose (pH 7.4 adjusted with NaOH). The pipette (4–6 MΩ) was filled with the intracellular solution (in mM): 140 CsCl, 5 EGTA, and 10 HEPES (pH 7.2 adjusted with CsOH). The action potential (AP) was evoked by a series of current pulses from 0 to + 180 pA with an increment of 20 pA.

### Immuno-histological assay

Paraffin-embedded human or mouse hippocampal brain tissues were sliced into 4-μm sections. For evaluating neuronal damage and loss after 4-AP insult, mouse hippocampal slices were performed Nissl staining and hematoxylin–eosin (HE) staining. Briefly, these sections were orderly deparaffinized and hydrated, and then stained, respectively, with toluidine blue solution (Biosharp, China, #BL999A) and HE solution (Biosharp, China, #BL700B). For assessing astrocyte reactivity after 4-AP insult, after antigen retrieval, the sections were blocked with 10% normal goat serum (Beyotime Biotechnology, China, #C0265) and then incubated overnight with GFAP antibody. On the second day, the sections were incubated with a horseradish peroxidase (HRP)-conjugated anti-mouse antibody and developed using DAB peroxidase substrate (Beyotime Biotechnology, China. #P0203). Localization and quantification of some proteins in human or mouse hippocampal tissues were performed similar immunofluorescence staining assay as primary cultured cells. For the immunofluorescence staining of frozen section, the mouse brain was sectioned at 15-μm thickness. Brain sections were washed with PBS for 5 min, three times, and then these sections were transferred to 0.3% Triton X-100 to incubate for 30 min at room temperature followed by blocking with 10% fetal sheep serum for 2 h. After incubation with the first antibody overnight at 4 ℃ and washing with PBS for 3 times, the slices were incubated in sequence with the corresponding secondary antibody for 2 h and DAPI for 10 min at room temperature. Immunofluorescence images were pictured using the confocal laser scanning microscope. The antibodies applied in immunohistochemical or immunofluorescent assay are listed in Table 1.

**Table 1:** Antibodies applied in immunohistochemical or immunofluorescent assay

	Manufacture	Dilution
Primary antibody		
Anti-NFIA	Abcam (ab228897)	1:1000 (WB)
		1:200 (IF)
Anti-TRPV4	Abcam(ab2040264)	1:500 (WB)
		1:200 (IF)
Anti-GFAP	CST(#3670)	1:1000 (WB)
		1:200 (IF)
		1:200 (IHC)
Anti-C3	Proteintech (21,337–1-AP)	1:1000 (WB)
		1:200 (IF)
		1:1000 (WB)
Anti-S100A10	Proteintech (11,250–1-AP)	1:200 (IF)
Anti-IL-6	Bioss(bs-0782R)	1:1000 (WB)
Anti-IL-1β	CST(#12242S)	1:500 (WB)
Anti-TNFα	ABclonal(A0277)	1:1000 (WB)
Anti-HA	CST(C29F4)	1:500 (WB)
Secondary antibody		
Goat anti-mouse IgG	Biosharp(BL001A)	1:10,000 (WB)
Goat anti-rabbit IgG	Biosharp(BL003A)	1:10,000 (WB)
HRP-conjugated anti-mouse antibody	Abbkine (A21010)	1:500 (IHC)

### Primary cell culture

Briefly, hippocampal tissues isolated from neonatal mice were performed trypsin enzymatic digestion and trituration and then the hippocampal cells were resuspended in complete medium (1 × Dulbecco’s modified Eagle’s medium (DMEM)/F12, 10% fetal bovine serum (FBS, Gibco, USA, #10,099-141), 1% L-glutamine, and 1% penicillin/streptomycin). For primary astrocyte culture, hippocampal cells were seeded on poly-D-lysine-coated Petri dishes and kept in an incubator (37 °C, 5% CO_2_). For primary neuron culture, the above cells were transferred in specific 19 neurobasal medium next day, while for primary microglia culture, the hippocampal cells were firstly seeded in T75 flasks and maintained in complete medium 12 days until they were isolated by vibrating 2 h at the speed of 200 rotation per minute. The medium was changed every 3 days to ensure adequate nutrition.

### Astrocytic transfection

The siRNA (si.NFIA or si.NC) and HA-tagged plasmid overexpressing NFIA (HA-NFIA) were transfected into primary astrocytes according to the protocol provided by the manufacturer (Polyplus, Strasbourg, France). Brief protocol for preparing the transfection mixture was as follows: the siRNA or plasmid was diluted in jetPRIME buffer and vortexed for 20 s, and then incubated for 10 min at room temperature. The transfection mixture was added in culture medium when astrocytes were in 60–70% confluence state.

### Fluorescent calcium imaging and quantification

Fluorescent calcium imaging was performed as previously described [18]. Briefly, primary cultured astrocytes were seeded in confocal dishes and were incubated with Fluo-4 AM (3 μM, Beyotime Biotechnology, China, #S1060) for 30 min at 37 °C in the dark, and then washed with PBS three times, subsequently followed by incubation in PBS for another 30 min at 37 °C, permitting Fluo-4 AM de-esterification and binding with cytosolic Ca^2+^. Confocal fluorescent calcium images of astrocytes were obtained by a confocal laser scanning microscope (Leica-LCS-SP8-STED, Leica, Wetzlar, Hesse, Germany) and real-time fluorescent calcium imaging was recorded. The ΔF/F0 ratio is calculated as (F–F0)/F0. Here, F0 is the basal fluorescence intensity of Fluo-4, while F represents the real-time fluorescence intensity before and after adding GSK1016790A (TargetMol, USA, #T6848), a specific TRPV4 agonist, and ΔF is the difference between F and F0.

### Immunofluorescence staining assay

Primary cultured cells were washed and fixed with 4% paraformaldehyde at room temperature for 30 min, and then permeabilized with Triton-X 100 for 20 min. Over simple incubation with 5% bovine serum albumin (BSA, Sigma-Aldrich, USA, #9048-46-8) for 30 min, they were transferred and kept overnight in the 5% BSA containing primary antibody at 4 °C. On the second day, after washing with PBS, the cells were orderly incubated with a corresponding secondary antibody for 1 h and followed by DAPI (labeling the nuclei) in the dark for 10 min at room temperature. Finally, immunofluorescence images were obtained using the confocal laser scanning microscope.

### Western blot measurement and density analysis

Proteins were extracted by homogenization in RIPA lysis buffer with phenylmethanesulfonyl fluoride. A BCA protein assay was used to quantify the protein concentration of the supernatants. Firstly, 30 μg protein for each lane was separated by sodium dodecyl sulfate–polyacrylamide gel electrophoresis and transferred to a polyvinylidene fluoride (PVDF) membrane. The PVDF membranes were then blocked with 5% BSA and incubated with primary antibodies (shown in Table 1) overnight at 4 °C. Afterwards, the membranes were washed with Tris-buffered saline containing 0.2% Tween-20 (TBST) and incubated with a corresponding horseradish peroxidase-conjugated secondary antibody. Then, the membranes were washed with TBST. Finally, the immunoreactive bands were detected by an enhanced chemiluminescence detection reagent. Band intensity was quantified by spot densitometric analysis using ImageJ software (version 1.41), and the results were normalized to GAPDH or β-actin levels and reported as the relative intensity of the control.

### Quantitative real-time PCR

TRIzol reagent (Vazyme, China, #R401) was used to extract total RNA from isolated mouse hippocampal tissues or primary cultured cells. Quantitative real-time polymerase chain reaction (qPCR) was performed with a SYBR Green Real-Time PCR master mix kit (Vazyme, China, #P111) according to the manufacturer’s instructions. The primers used in this study are shown in Table 2 and the expression of target genes was presented as the fold change normalized to the mRNA level of the internal control β-actin. The 2^−ΔΔCt^ relative quantification method was used to analyze the expression of target genes.

**Table 2:** q-PCR primer sequences

Primer	Froward primer 5′-3′	Reverse primer 5′-3′
*β-actin*	CACGATGGAGGGGCCGGACTCAT	TAAAGACCTCTATGCCAACACAGT
*NFIA*	GAACTTGGTGGATGGATGAGTT	CACTTCTGCTTGACCTCGGG
*TRPV4*	GAACTTGGTGGATGGATGAGTT	CACTTCTGCTTGACCTCGGG

### Chromatin immunoprecipitation (ChIP)

The ChIP assay (Beyotime Biotechnology, China, #P2078) was performed as previously described [21]. Astrocytes were cross-linked in PBS containing 1% formaldehyde for 10 min at room temperature and quenched with 500 μl 2.5 M glycine. After crosslinking, the cells were washed with PBS twice and resuspended in 1 ml lysis buffer (50 mM Tris–HCl, pH 8, 0.5% SDS and 5 mM EDTA) for 10 min at 4 °C. The lysates were subjected to sonication to obtain 200- to 500-bp fragments of DNA (10 min cycle, 5 s pulses; amplitude, 30%) and then centrifuged at 12,000*g* at 4 °C for 10 min to obtain the supernatants. Next, 10% of the supernatants were kept as input samples, and the remaining samples were divided according to the antibodies (against NFIA and IgG). Each sample was diluted 1:4 with dilution buffer (20 mM Tris–HCl, pH 8.0, 150 mM NaCl, 2 mM EDTA and 1% Triton X-100). The samples were precleared with pretreated protein A or G beads (1 mg/ml BSA), 1 mg/ml sperm DNA and 20% beads) for 2 h at 4 °C. The aliquots were then incubated overnight at 4 °C with pretreated protein A or G beads and antibodies (against NFIA and IgG). After extensive washing (four times each) with RIPA buffer, wash buffer (20 mM Tris–HCl, pH 8.0, 1 mM EDTA, 250 mM LiCl, 0.5% NP-40 and 1 mM MSF) and TE buffer (10 mM Tris–HCl, pH 8.0 and 1 mM EDTA), the beads were resuspended in TE buffer. The resuspended beads were subjected to RNase A and proteinase K digestion, and the crosslinking was reversed at 65 °C for 8–10 h. DNA was recycled with a DNA purification kit.

### Statistical analysis

All data are presented as the mean ± standard error of the mean. GraphPad Prism v7.00 (GraphPad, La Jolla, CA, USA) was used to analyze the data and construct the graphs. Student’s *t* test and one-way analysis of variance (ANOVA) were performed to determine the significant differences between groups. For multiple groups, one-way ANOVA was used for statistical analysis. For in vivo experiments, the n values refer to the number of individual animals in each group. For in vitro studies, the n values indicate the number of times the experiment was independently replicated in cultures derived from different mice, as indicated in the figure legends. For immunofluorescent intensity analysis of human specimen, the specimens were numbered randomly and the average fluorescent intensity of three visual fields (0.5 mm^2^ per visual field) in each specimen was quantified blindly. No data was excluded. *P* < 0.05 indicated that the difference was statistically significant.

## Results

### NFIA knockdown attenuates 4-AP-induced seizure and neuronal damage

We firstly injected si.NFIA or si.NC in mouse hippocampal cortex in vivo, and killed these mice at 24, 48, and 72 h, respectively. Western blot was used to detect the protein levels of NFIA in the mouse hippocampal brain tissues (Additional file 1: Fig. S1A). The results showed that NFIA protein expression markedly falls down in those mice treated with si.NFIA 72 h (Additional file 1: Fig. S1B).

**Fig. 1:**
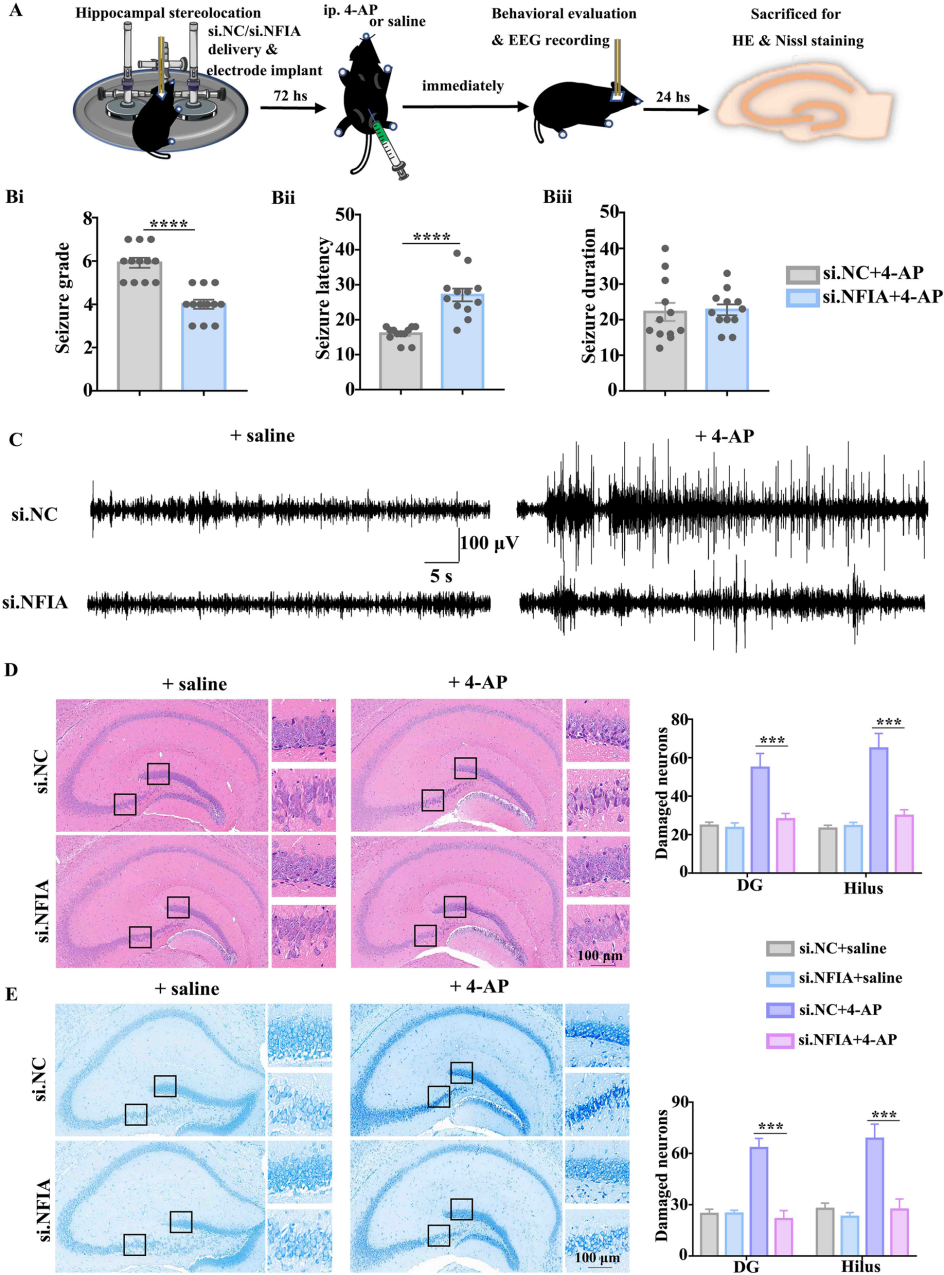
Knockdown of NFIA attenuates 4-AP-induced seizure and neuronal damage. **A** Scheme of in vivo and in vitro experiments in 4-AP-induced seizure mouse model. Behavioral evaluation showing 4-AP-induced seizure grade (**Bi**), seizure latency (**Bii**) and seizure duration (**Biii**) in mice with hippocampal si.NC or si.NFIA delivery (*n* = 12 for each group, ****P < 0.0001, Student’s *t* test). Representative EEG recordings (**C**) in mice with ip. 4-AP or saline (*n* = 6 for each group). **D** HE and Nissl **E** staining in the hippocampal tissues from the mice with si.NC/si.NFIA delivery and ip. 4-AP/saline, especially showing darker staining in DG and hilus, with cytoplasmic shrinkage, and triangulated pyknotic nuclei in si.NC group, but not in the si.NFIA group (*n* = 6 for each group, ***P < 0.001, one-way ANOVA)

4-AP is a blocker of voltage-gated potassium channels and commonly used to induce acute epileptic seizure events in animal model in vivo and in vitro [18, 22–24]. We asked whether the alteration of NFIA expression affects 4-AP-induced seizure activities and neuronal damage in mouse model. The mice were subjected to EEG recording electrode implantation immediately after hippocampal si.NFIA or si.NC delivery; 72 h later, the mice were injected intraperitoneally either 4-AP to induce an epileptic seizure, or saline as a control (Fig. 1A). Behavioral observation showed that seizures are alleviated in the si.NFIA-treated mice, as evidenced by the significant decrease in seizure grade and prolongation of seizure latency, even though no apparent change in seizure duration is detected (Fig. 1B). EEG analysis showed that 4-AP-induced aberrant discharges are profoundly inhibited in the si.NFIA-treated mice compared with the si.NC group (Fig. 1C). 24 h later, these intraperitoneal 4-AP or saline mice were killed to perform HE staining (Fig. 1D) and Nissl staining (Fig. 1E). Both HE and Nissl staining results showed that 4-AP-induced neuronal morphology changes such as cytoplasmic shrinkage and triangulated pyknotic nuclei are remarkable in dentate gyrus (DG) and hilus in the mice with hippocampal si.NC injection, but rare in those with hippocampal si.NFIA injection. These findings suggest that the deficiency of NFIA alleviates 4-AP-induced seizures and reduces neuronal damage.

### NFIA knockdown prevents hippocampal neurons from increased excitation induced by 4-AP

**Fig. 2:**
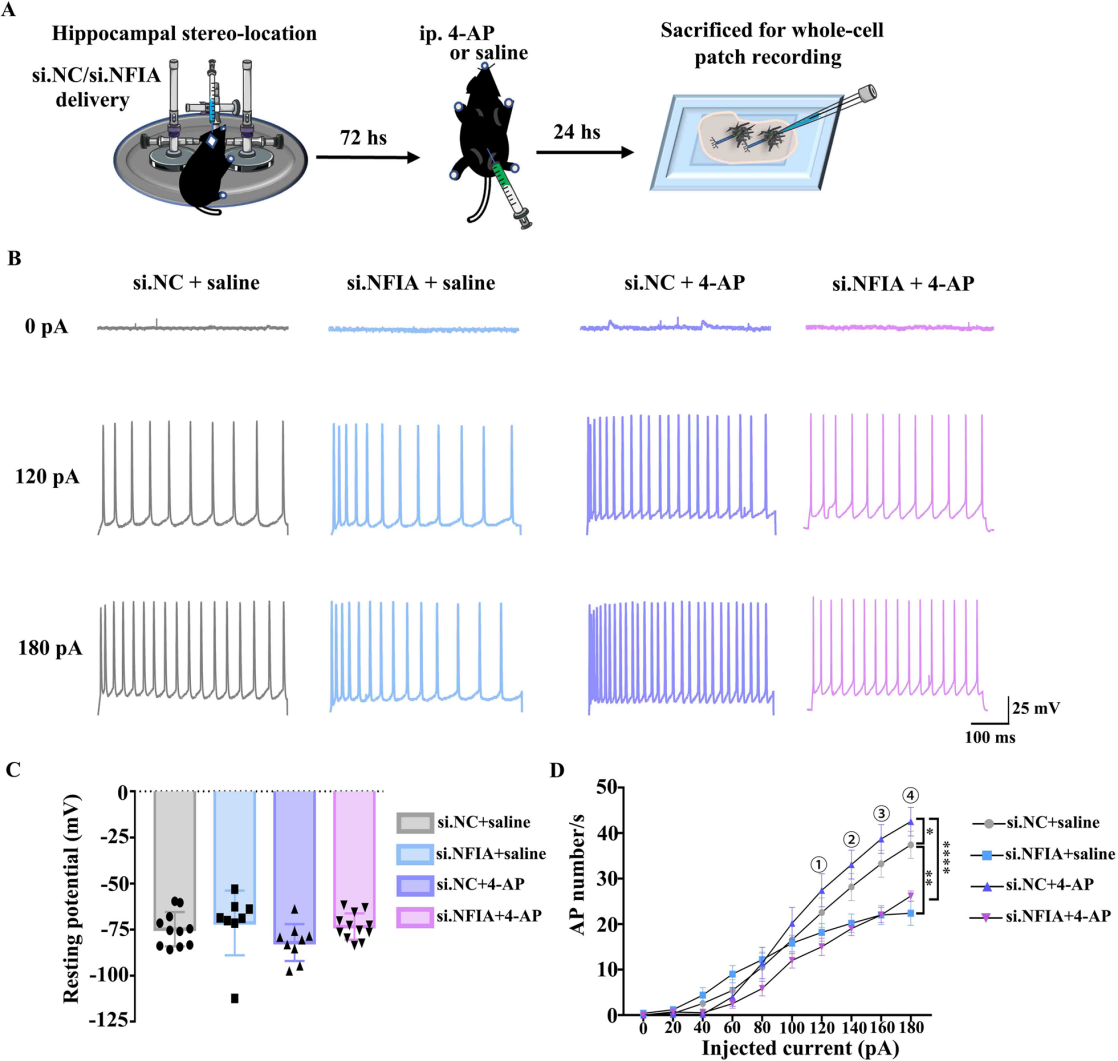
NFIA knockdown prevents hippocampal neurons from 4-AP increased excitation. **A** Scheme of animal preparation for whole-cell patch recording experiment. Representative curves (**B)** of neuronal AP discharge evoked by step currents from 0 to 180 pA with 20 pA increment in whole-cell recording and the analysis of resting membrane potential (**C)** and AP number (**D)** of CA1 pyramidal neurons in hippocampal slices from the mice with si. NC/si. NFIA delivery and ip. 4-AP/saline (si.NC: *n* = 11, si.NFIA: *n* = 8, si.NC + 4-AP: *n* = 9, si.NFIA + 4-AP: *n* = 12, *P < 0.05, **P < 0.01, ****P < 0.0001, One-way ANOVA)

Although 4-AP evoked a single generalized seizure and damaged partial neurons, survival neurons frequently exhibited higher firing rate upon current stimulation and were more likely to trigger another seizure [18, 25]. To evaluate whether in vivo si.NFIA treatment alters hippocampal neuronal excitability upon stimulation, the mice with hippocampal si.NC or si.NFIA treatment 72 h were intraperitoneally injected with saline or 4-AP, and killed over another 24 h to prepare brain slice for detecting the electrical activities of CA1 hippocampal pyramidal neurons by whole-cell patch recording (Fig. 2A).

No significant change at resting membrane potential is observed in mouse hippocampal pyramidal neurons from four groups (Fig. 2C), and the neuronal AP number appears gradient increase upon step current stimulation in each group (Fig. 2B). However, in si.NC-treated mice, the pyramidal neurons after 4-AP insult exhibit greater AP number than those neurons in saline group (Fig. 2B, D). By contrast, in si.NFIA-treated mice, although the pyramidal neurons after 4-AP insult exhibit a tendency of slight increase in AP firing number compared with those neurons in saline group, no remarkable difference exists between two groups (Fig. 2B, D). Furthermore, under the same insult from 4-AP, the AP firing number evoked by each current ranging from 120 to 180 pA is markedly lower in the pyramidal neurons from si.NFIA-treated mice than in those neurons from si.NC-treated mice (Fig. 2D), suggesting that knocking down NFIA in hippocampal tissues in advance can vastly prevent pyramidal neurons from 4-AP-evoked increase of excitation upon current stimulation. The above in vitro and in vivo electrophysiological examinations together with behavioral evaluation indicate that NFIA may be a key factor in 4-AP-induced epileptic seizure.

### 4-AP-induced NFIA upregulation is cell-specific in mouse hippocampal astrocytes

**Fig. 3:**
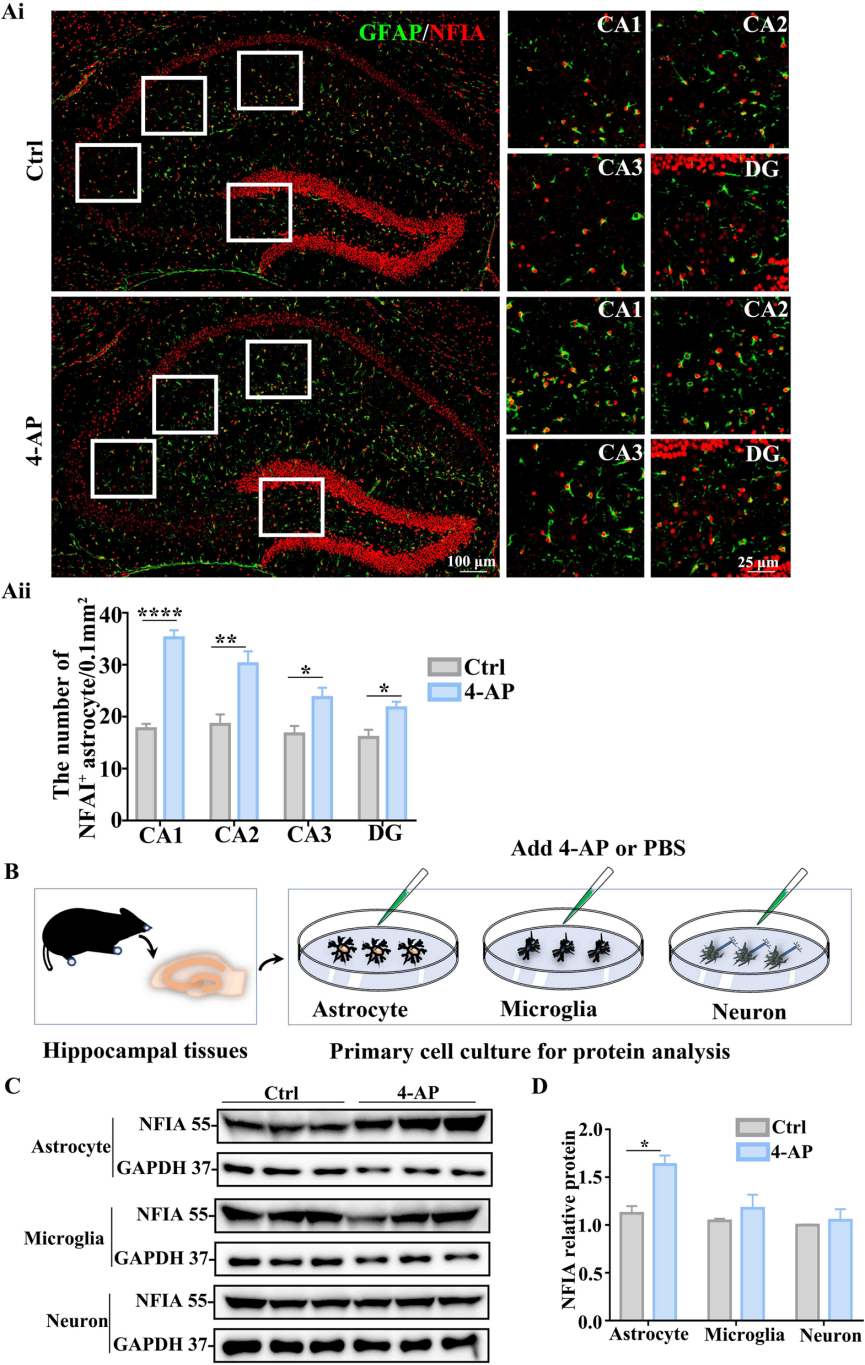
4-AP-induced NFIA upregulation is cell-specific in mouse hippocampal astrocytes. Representative confocal fluorescent images of NFIA^+^ astrocytes (**Ai**) in hippocampal tissues and quantitative analysis of NFIA^+^ astrocytes in CA1, CA2, CA3 and DG (**Aii**) from the mice with ip. 4-AP or saline (*n* = 6 for each group, *P < 0.05, Student’s *t* test)*.*** B** Scheme of primary astrocyte, microglia, neuron culture and 4-AP exposure for protein analysis. Representative immunoblot bands (**C**) and protein level analysis (**D**) of NFIA in three types of primary cultured cells treated with 4-AP or saline (*n* = 3 for each group, *P < 0.05, Student’s *t* test)

NFIA is known as a molecular switch for inducing human glial competency and NFIA-induced astrocytes promote synaptogenesis in the adult mouse brain [15], and NIFA is also expressed in neurons [26]. We sought to determine whether the effect of NFIA on 4-AP-evoked hippocampal neuronal excitation is cell-specific, thus, we performed triple immunofluorescence assays to observe the expression alteration of NFIA in hippocampal region in the mice with intraperitoneal treatment of 4-AP or saline 24 h. The immunofluorescence assay results showed that the increase of 4-AP-induced mouse NFIA^+^ cell number is observed in astrocytes (Fig. 3A), barely in neurons and microglia (Additional file 1: Fig. S2), whether in CA1, CA2, CA3 or in DG. These immunofluorescence assay results hint that the phenomenon of more susceptible to excitation in those hippocampal neurons after 4-AP insult may be attributed to the upregulation of astrocyte-specific NFIA expression.

Next, we isolated mouse hippocampal tissues to perform primary cultures of astrocyte, microglia and neuron, and treated these three types of cells with 4-AP 24 h or PBS as a control (Fig. 3B). Protein analysis from primary cultured cells exhibited that 4-AP-induced NFIA upregulation only occurs in astrocytes, neither in microglia nor in neurons (Fig. 3C, D), which is consistent with in vivo hippocampal NFIA expression in the mice with 4-AP treatment. Moreover, in line with upregulation of NFIA and GFAP, 4-AP-induced hippocampal astrocytes present enlarged cell body and increased branches, suggesting that the astrocyte reaction in the presence of 4-AP may be closely related with NFIA upregulation.

### NFIA knockdown reverses 4-AP-induced hippocampal astrocyte state transition

**Fig. 4:**
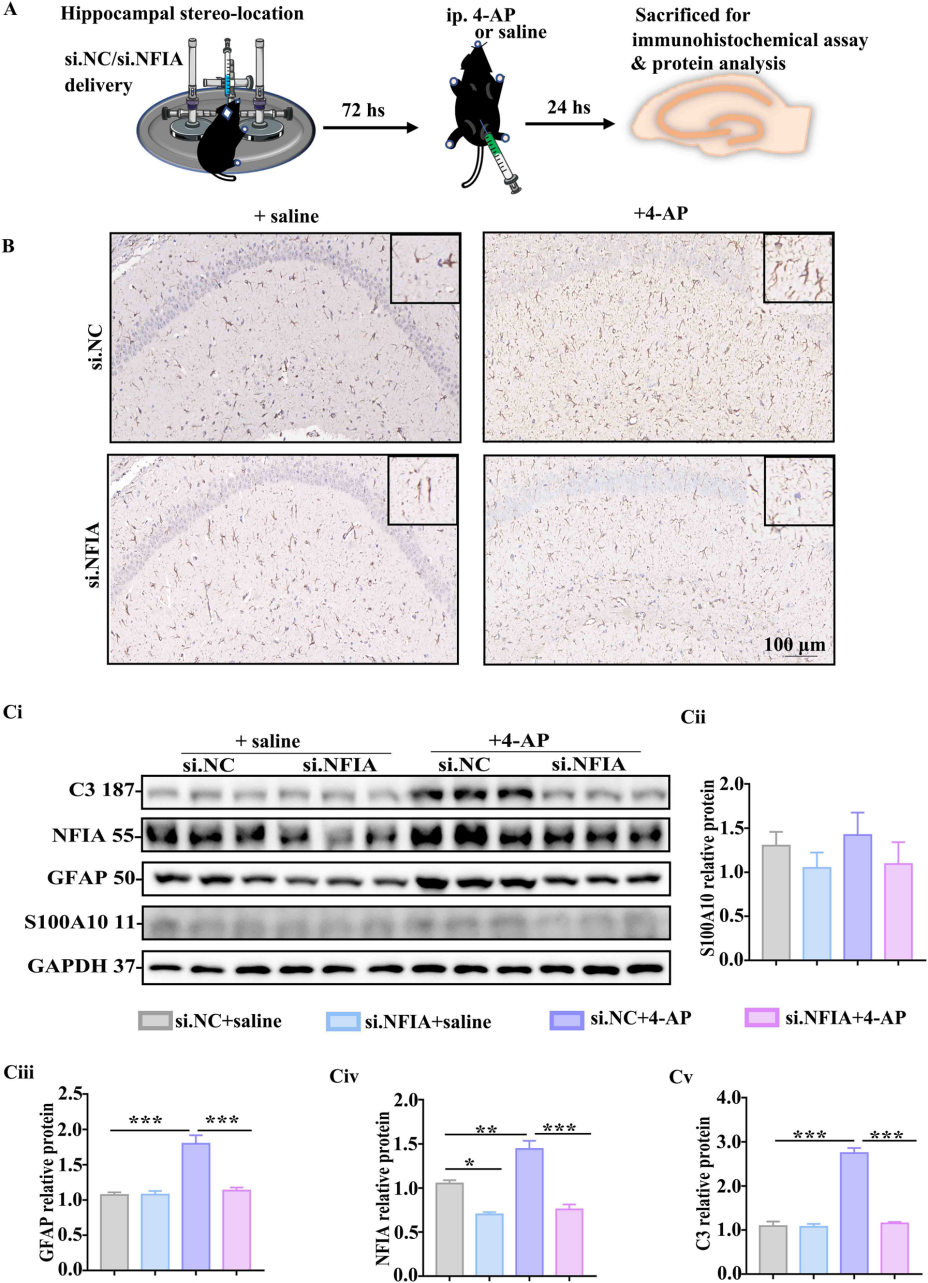
NFIA knockdown reverses 4-AP-induced hippocampal astrocyte state transition. **A** Scheme of animal preparation for immunohistochemical assay and protein analysis. **B** Immunohistochemical staining of GFAP in the hippocampal tissues from the mice with si.NC/si.NFIA delivery and ip. 4-AP/saline. **Ci** Representative immunoblot bands of S100A10, GFAP, NFIA and C3 in hippocampal tissues from the mice with si. NC/si. NFIA delivery and ip. 4-AP/saline, and the band density analysis of **Cii** S100A10, **Ciii** GFAP, **Civ** NFIA, and **Cv** C3 (*n* = 3 for each group, *P < 0.05, **P < 0.01, ***P < 0.001, One-way ANOVA)

Astrocytes after insult may undergo diverse state transition in the CNS disorders, characterized by different morphology and molecular markers. We sought to test whether NFIA knock-down impacts the state transition of astrocytes by detecting the expression of some specific markers of reactive astrocytes both in hippocampal tissues from the mice with hippocampal si.NC or si.NFIA delivery (Fig. 4A) and in primary cultured astrocytes transfected with si.NC or si.NFIA (Additional file 1: Fig. S3A). The immunohistochemistry analysis showed that 4-AP-induced reactive astrocytes with enlarged cell body and increased branches are only observed in si.NC group, almost not present in si.NFIA group (Fig. 4B). Western blot results showed that 4-AP induced elevation of astrocytic GFAP and the third component of complement (C3), mainly exist in the mice treated with si.NC, nearly not in si.NFIA group (Fig. 4C), while S100A10 immunolabeling astrocyte appears no apparent change in the mice with si.NC or si.NFIA delivery, regardless of 4-AP treatment (Fig. 4C). In primary cultured astrocytes, si.NFIA transfection 48 h can efficiently inhibit NFIA expression compared with si.NC group (Additional file 1: Fig. S3C). Responding to 4-AP exposure for 24 h, hippocampal astrocytes transfected with si.NFIA show weaker fluorescence intensity of GFAP and C3 (Additional file 1: Fig. S3B) and lower density of GFAP and C3 in western blot bands (Additional file 1: Fig. S3C) than those cells in si. NC group, while no difference in S100A10 expression exists in hippocampal astrocytes transfected with si.NC or si.NFIA, whether 4-AP is added or not (Additional file 1: Fig. S3C). These in vivo and in vitro experimental results indicate that NFIA plays a critical role in 4-AP-induced proinflammatory state transition of reactive astrocytes and NFIA knock-down can prevent this transition.

Our recent study showed that blocking TRPV4 can mitigate 4-AP-induced epileptogenesis in mice by preventing astrocytes from converting to be proinflammatory state [18]. Another study proved that reactive astrocytes may exert pro-seizure via enhancing calcium activity [27]. Therefore, we ask whether calcium-permeable cation channel TRPV4 is the main effector molecule of NFIA in hippocampal reactive astrocyte after 4-AP insult.

### Both NFIA and TRPV4 are upregulated in TLE patients and 4-AP-induced seizure mouse model

**Fig. 5:**
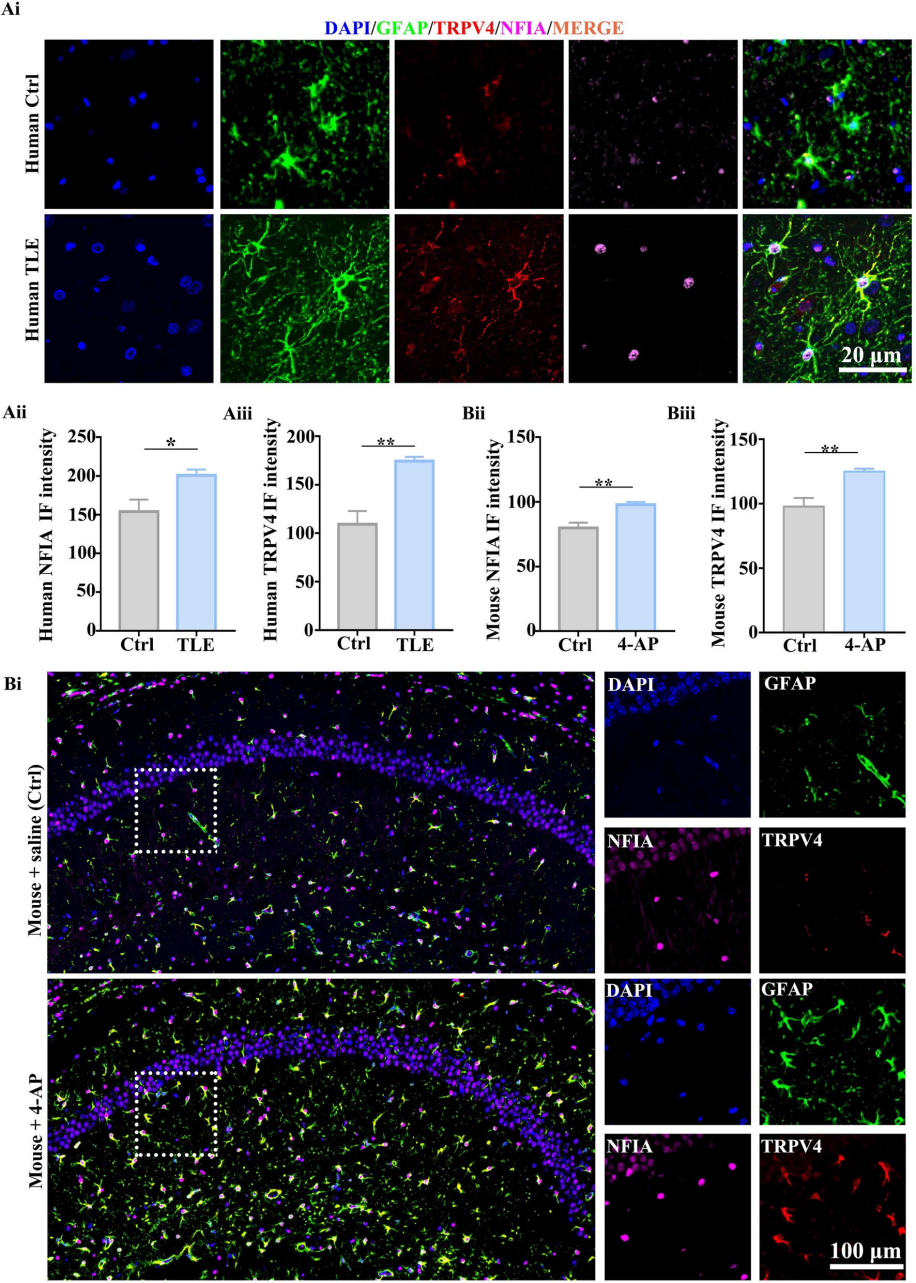
Astrocytic NFIA and TRPV4 expression are upregulated in TLE patients and 4-AP-induced seizure mouse model. Representative confocal images (**Ai)** of TRPV4 and NFIA in hippocampal tissues from the TLE patients who had surgery and the deceased without TLE as a control, and three visual fields (0.5 mm^2^ per visual field) in each human specimen were applied for the fluorescence intensity analysis of **Aii** NFIA and **Aiii** TRPV4 (human TLE: *n* = 5, Human Ctrl: *n* = 6, * *P* < 0.05, **P < 0.01, Student’s *t* test)*.* Representative confocal immunofluorescent images **Bi** of TRPV4 and NFIA, and the fluorescence intensity analysis of **Bii** NFIA and **Biii** TRPV4 in hippocampal tissues from the mice with ip. 4-AP or saline (*n* = 6 for each group, **P < 0.01, Student’s *t* test)

Firstly, we performed multiple immunofluorescent staining assay to detect the expression of GFAP, NFIA and TRPV4 in human hippocampal tissues from the TLE patients who had surgery and the deceased who had postmortem (control). Compared with the control group, majority of astrocytes in the brain tissues from surgical patients with TLE have more and longer branches (Fig. 5A), with a concomitant increase of NFIA and TRPV4 in immunofluorescent intensity (Fig. 5A). Next, we performed identical staining in hippocampal tissues from the mice with intraperitoneal 4-AP or saline as a control. As expected, in hippocampal region from the mice with 4-AP treatment, larger cell body and more branches are observed in a mass of astrocytes with increased GFAP immunofluorescent intensity (Fig. 5B); meanwhile, both NFIA and TRPV4 immunofluorescent intensity is also increased in these reactive astrocytes (Fig. 5B). In addition, qPCR and western blot analysis showed that the mRNA and protein levels of NFIA and TRPV4 are increased in hippocampal tissues from the mice with 4-AP intraperitoneal injection (Additional file 1: Fig. S4A, B). Similar results on the expression of above molecules in primary cultured astrocytes are found in 4-AP-induced seizure cell model (Additional file 1: Fig. S4C–E). These findings suggest that consistent upregulation of NFIA and TRPV4 may be the common mechanisms in the development of human TLE and mouse 4-AP-induced seizure.

### Astrocytic NFIA knockdown prevents TRPV4 upregulation and its calcium activity

Given the coincident expression alteration of these three proteins (GFAP, NFIA and TRPV4) in hippocampal tissues from human patients with TLE and mice with 4-AP-induced seizure, we speculate that NFIA can upregulate TRPV4 to promote astrocyte reactivity. TRPV4 protein level in hippocampal tissue from mice with si.NFIA treatment is lower than that in the si.NC group (Additional file 1: Fig. S5). Although TRPV4 is constitutively expressed both in astrocytes and neurons from human and mouse hippocampal tissues, 4-AP-induced TRPV4 upregulation only occurs in astrocytes, not in neuron [18]. Next, to better understand the mechanisms that cell-specific NFIA upregulation controls astrocyte reactivity in 4-AP-induced seizure model, we test the effect of NFIA knockdown on the expression of TRPV4 and its functional activity in primary cultured astrocytes.

**Fig. 6:**
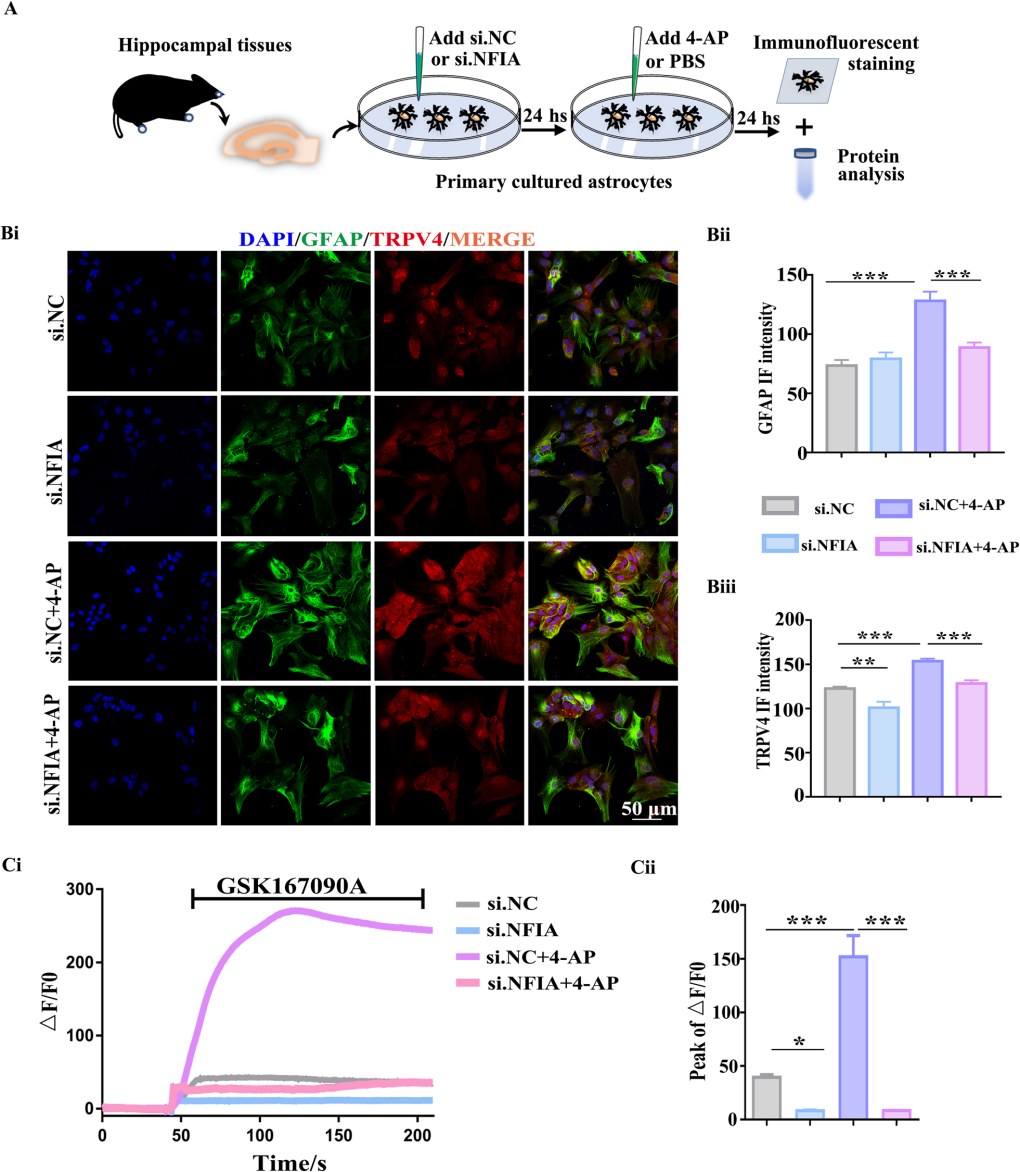
Astrocytic NFIA knockdown prevents TRPV4 upregulation and its calcium activity. **A** Scheme of primary astrocyte preparation for immunofluorescent staining and protein analysis. Representative confocal immunofluorescent images (**Bi)** of GFAP and TRPV4, and the immunofluorescent intensity analysis of GFAP (**Bii**) and TRPV4 (**Biii**) in the astrocytes transfected with si.NC or si.NFIA in the presence or absence of 4-AP (si.NC: *n* = 7, si.NFIA: *n* = 6, si.NC + 4-AP: *n* = 6, si.NFIA + 4-AP: *n* = 6, **P < 0.01, ****P < 0.001, One-way ANOVA)*.* **Ci** Representative traces of Ca^2+^ indicator Fluo-4 fluorescence intensity changes in astrocytes responding to GSK1016790A, and the analysis (**Cii**) of the increased Fluo-4 fluorescence intensity peak, ΔF/F0, which was calculated as (F–F0)/F0. F0 is the mean value of fluorescent intensity before the addition of GSK1016790A (*n* = 30 cells for each group, *P < 0.05, ***P < 0.001, one-way ANOVA)

We performed identical experimental procedure (Fig. 6A) to detect the influence of NFIA knockdown on TRPV4 expression and TRPV4-mediated calcium signal, respectively. Immunofluorescent staining assay showed that 4-AP can induce the increase of TRPV4 and GFAP immunofluorescent intensity in si.NC group, but nearly not in si.NFIA group (Fig. 6B). Accordingly, protein analysis exhibited that 4-AP-induced TRPV4 and NFIA upregulation exists in si.NC group and control group without siRNA transfection, but not in si.NFIA group (Additional file 1: Fig. S6A). In line with TRPV4 expression, confocal imaging results (Additional file 1: Fig. S6B) showed that the fluorescent intensity of Fluo-4, a Ca^2+^ indicator, is lower in the astrocytes transfected with si.NFIA than that in si.NC group, even under resting state. 4-AP exposure remarkably increases Fluo-4 fluorescent intensity in si.NC-transfected astrocytes, but fails to increase Fluo-4 fluorescent intensity in those cells transfected with si.NFIA. Similarly, real-time calcium imaging analysis (Fig. 6C) showed that in si.NC-transfected astrocytes, the increased Fluo-4 fluorescent intensity triggered by GSK1016790A, a specific agonist of TRPV4, is significantly greater in the presence of 4-AP than that in the absence of 4-AP, while almost slight increase of Fluo-4 fluorescent intensity triggered by GSK1016790A is observed in si.NFIA transfected astrocytes, regardless of 4-AP. These results hint that NFIA may affect cytosolic calcium level by controlling astrocytic TRPV4 quantity.

### Astrocytic NFIA transcriptionally regulates TRPV4 expression by direct binding to its promoter

**Fig. 7:**
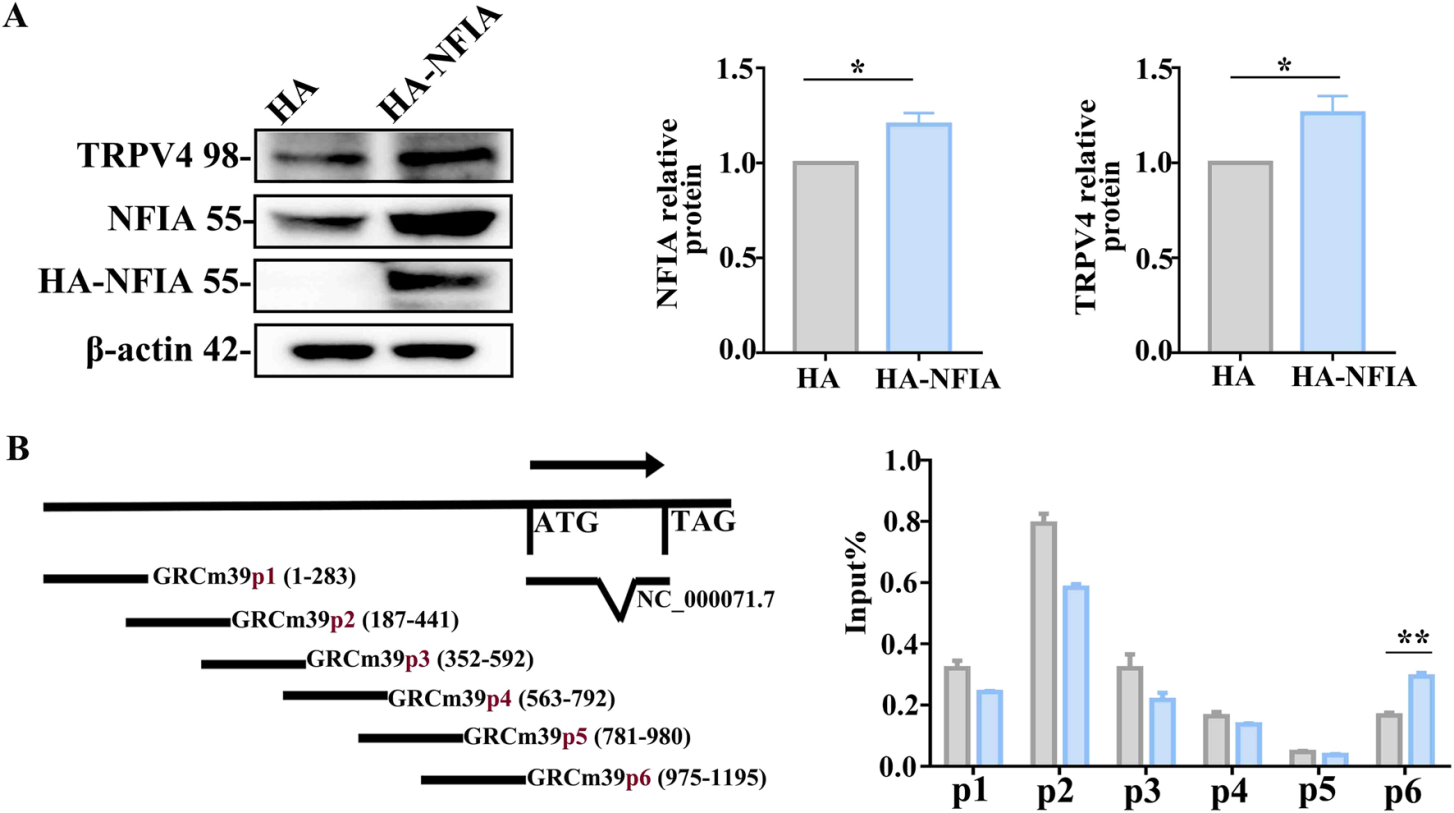
Astrocytic NFIA transcriptionally regulates TRPV4 expression by direct binding to its promoter.** A** Representative immunoblot bands of NFIA and TRPV4 in primary cultured astrocytes transfected with NFIA overexpression plasmid (HA-NFIA) or vector plasmid (HA) and their density analysis (*n* = 3 for each group, *P < 0.05, Student’s *t* test). **B** ChIP assays showing binding of NFIA and TRPV4 promoter: left, the primer sets at the TPRV4 promoter; right, the qPCR analysis of the enrichment region of NFIA at the TRPV4 promoter sequence (*n* = 3, **P < 0.01, Student’s *t* test)

Next, we asked whether NFIA as transcriptional factor directly regulates TRPV4 expression in astrocytes. We constructed an HA-NFIA plasmid and six pairs of primers targeting the TRPV4 promoter regions (upstream 0~1200 bp) to track NFIA binding sites in TRPV4 gene using ChIP assay. Protein analysis showed that NFIA expression is augmented in primary cultured astrocytes transfected with HA-NFIA plasmid 24 h, with a concomitant increase of TRPV4 (Fig. 7A). The qPCR analysis in the ChIP experiment showed that NFIA is specifically concentrated at GRCm39p6 of TRPV4 gene (Fig. 7B), suggesting direct binding of NFIA with the promoter region 975~1195 of TRPV4.

### Functional TRPV4 is required for NFIA-regulated astrocyte state transition

**Fig. 8:**
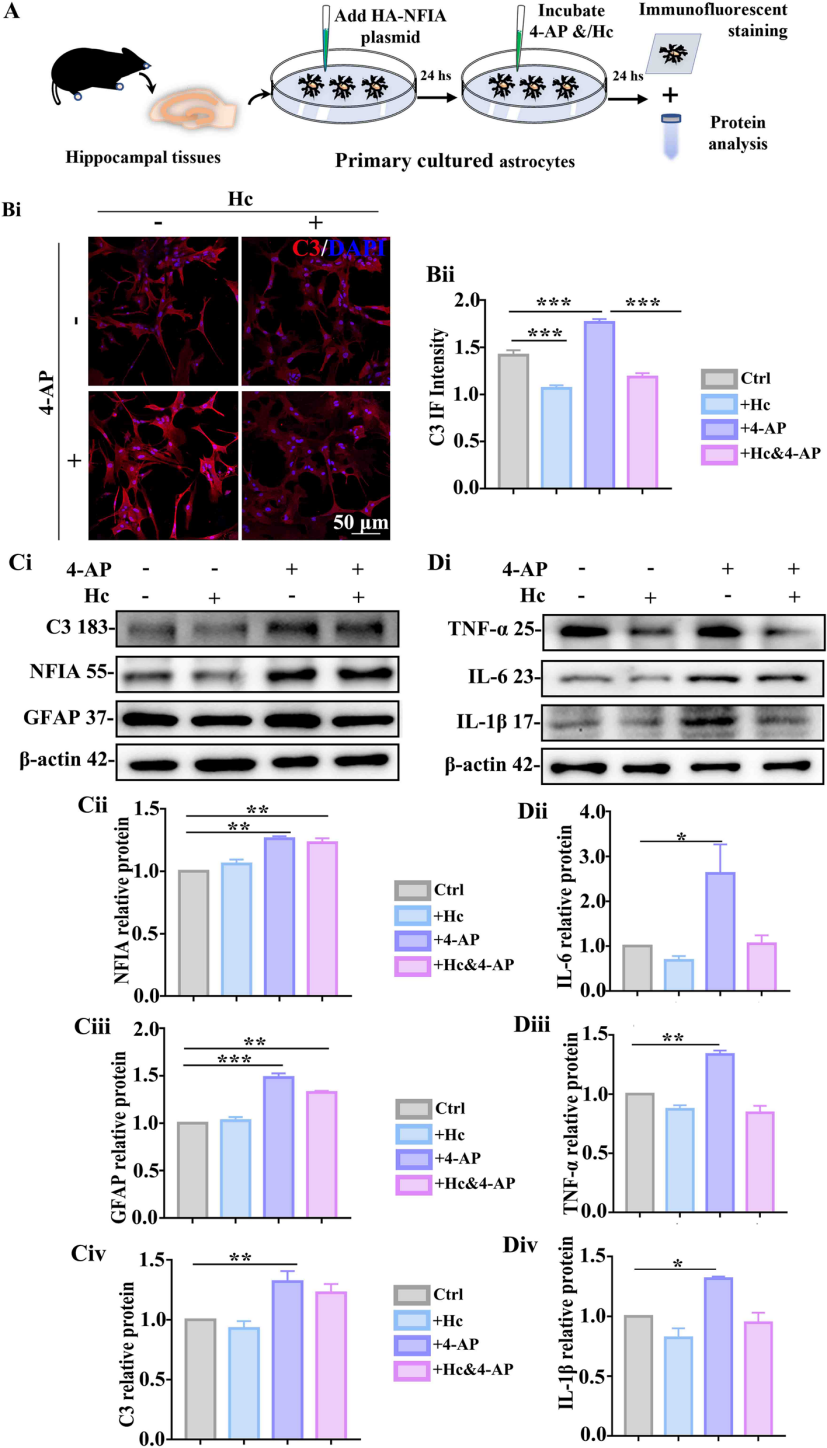
Functional TRPV4 is required for NFIA-regulated astrocyte conversion. **A** Schema of overexpressing HA/HA-NFIA astrocyte preparation for exploring the role of TRPV4 functional activity. Representative confocal immunofluorescent images (**B**i) of C3 and its immunofluorescent intensity analysis (**Bii**) in NFIA-overexpressing astrocytes with/without 4-AP exposure in the presence or absence of Hc, a TRPV4 antagonist (*n* = 6 for each group, ***P < 0.001, one-way ANOVA)*.* (**Ci**) Representative immunoblot bands of NFIA, GFAP and C3, and the band density analysis of NFIA (**Cii**), GFAP (**Ciii**) and C3 (**Civ**) in NFIA-overexpressing astrocytes with/without 4-AP exposure in the presence or absence of Hc (*n* = 3 for each group, **P < 0.01, ***P < 0.001, one-way ANOVA)*.* (**Di**) Representative immunoblot bands of TNF-α, IL-6 and IL-1β, and the band density analysis of IL-6 (**Dii**), TNF-α (**Diii**) and IL-1β (**Div**) in NFIA-overexpressing astrocytes with/without 4-AP exposure in the presence or absence of Hc (*n* = 3 for each group, **P < 0.01, ***P < 0.001, One-way ANOVA)

Given the important role of NFIA in TRPV4 transcriptional regulation and proinflammatory astrocyte reaction in 4-AP-induced seizure, with our previous study on the effects of TRPV4 on 4-AP-mediated astrocyte reactivity and neuroinflammation [18], we sought to detect whether the astrocyte reactivity depends on the functional activity of TRPV4. Next, we utilized HC-067047 (TargetMol, USA, #T4680), a specific TRPV4 antagonist, to test the effects of blocking TRPV4 on the expression of proinflammatory astrocyte markers and some inflammatory cytokines in primary cultured astrocytes transfected HA-NFIA plasmid (Fig. 8A). Immunofluorescent staining results showed that in the absence of HC-067047, the enhanced immunofluorescent intensity of C3 is observed in the astrocytes with 4-AP treatment; nevertheless, this increase almost disappears in the presence of HC-067047 (Fig. 8B). Protein analysis showed that 4-AP induces NFIA upregulation even in the astrocytes transfected with HA-NFIA plasmid, accompanied by significant GFAP upregulation and mild C3 upregulation; likewise, 4-AP-induced mild C3 upregulation almost completely vanishes in the presence of HC-067047 (Fig. 8C). In addition, in the absence of HC-067047, proinflammatory cytokines such as tumor necrosis factor-α (TNF-α), interleukin (IL)-6 and IL-1β are also upregulated in the astrocytes treated with 4-AP (Fig. 8D). The addition of HC-067047 instead inhibits 4-AP-induced upregulation of these cytokines. The above results disclose that the functional activity of TRPV4 is required for the astrocyte reactivity mediated by NFIA-TRPV4 signaling.

### Hippocampal astrocytic NFIA knockdown attenuates 4-AP-induced seizure and neuronal damage

**Fig. 9:**
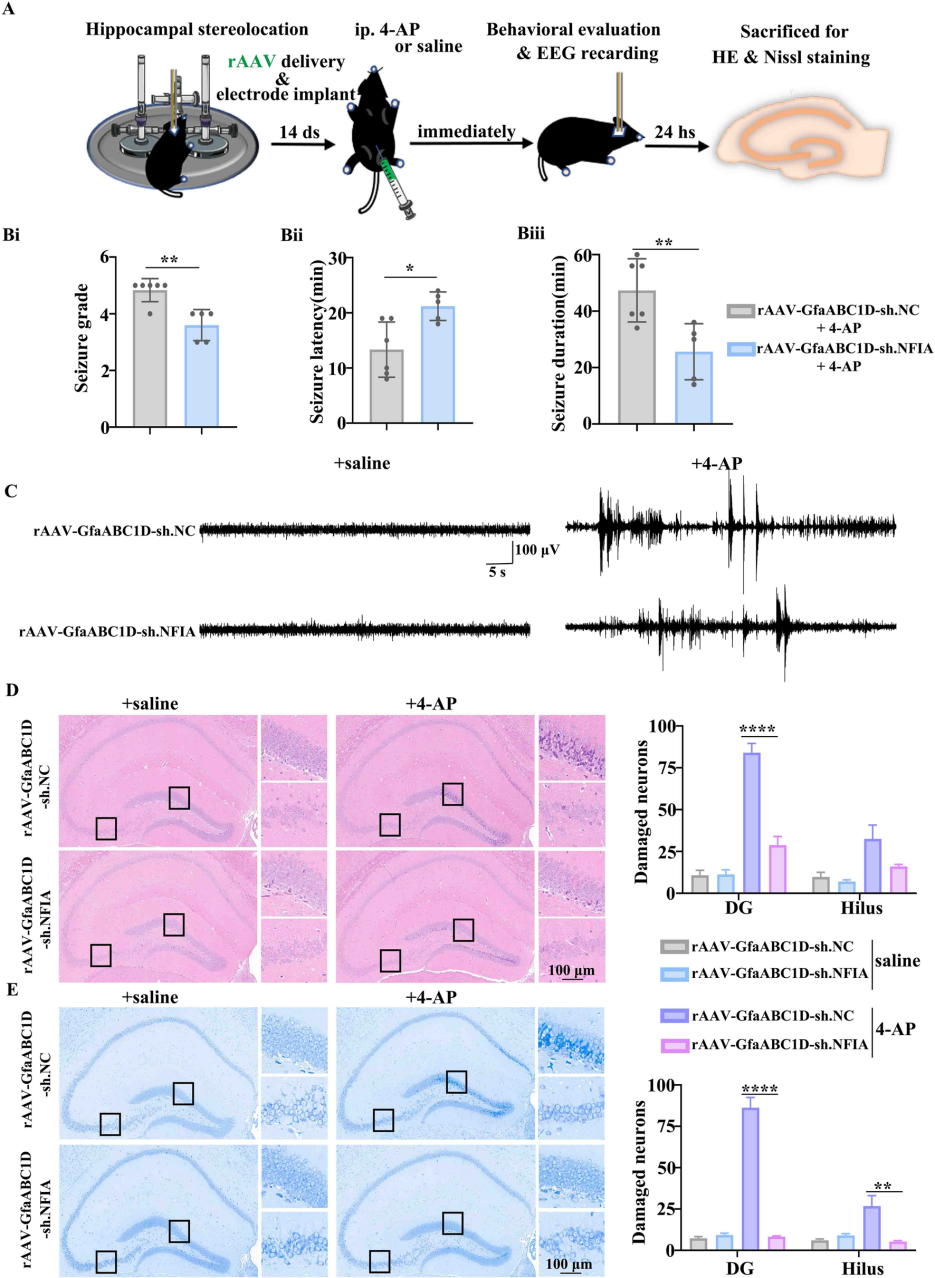
Effects of hippocampal astrocyte-specific NFIA knockdown on 4-AP-induced seizure and neuronal damage in mice.** A** Scheme of experiments in 4-AP-induced seizure mouse model. 4-AP-induced behavioral observation including **Bi** seizure grade, **Bii** seizure latency and **Biii** seizure duration in mice with hippocampal delivery of rAAV- GfaABC1D-sh.NC (n = 6) or rAAV-GfaABC1D-sh.NFIA (*n =* 5), *P < 0.05, **P < 0.01, Student’s *t* test. Representative EEG recordings (**C**) in mice infected with rAAV-GfaABC1D-sh.NC (*n =* 6 for both ip. saline and ip. 4-AP group) or rAAV-GfaABC1D-sh.NFIA (*n =* 5 for both ip. saline and ip. 4-AP group). HE (**D**) and Nissl (**E**) staining in the hippocampal tissues from the mice infected with rAAV-GfaABC1D-sh.NC (*n =* 6 for both ip. saline and ip. 4-AP group) or rAAV-GfaABC1D-sh.NFIA (*n =* 5 for both ip. saline and ip. 4-AP group), showing 4-AP-induced neuronal damage in DG and hilus from the mice infected with rAAV-GfaABC1D-sh.NFIA is significantly alleviated compared with that in rAAV-GfaABC1D-sh.NC-infected group, **P < 0.01, ****P < 0.0001 one-way ANOVA

To further determine the critical role of astrocyte-specific NFIA in 4-AP-induced epileptic seizure, rAAV-GfaABC1D-sh.NFIA was firstly injected into the hippocampal cortex in the mice to specifically knock down the expression of NFIA in hippocampal astrocytes while rAAV-GfaABC1D-sh.NC was used as control (Additional file 1: Fig. S7A, Fig. 9A), 14 days later, immunofluorescence assay was performed to detect the interference efficiency of astrocytic NFIA (Additional file 1: Fig. S7A), and then 4-AP-induced seizure mouse model was established to perform in vivo behavioral observation and EEG recording and in vitro tissue staining (Fig. 9A). The result of immunofluorescent assay showed that red immunofluorescence of NFIA is clearly visible in hippocampal astrocytes infected with rAAV-GfaABC1D-sh.NC but almost completely absent in hippocampal astrocytes infected with rAAV-GfaABC1D-sh.NFIA (Additional file 1: Fig. S7B). Behavioral evaluation showed that 4-AP-induced seizure grade is decreased and seizure duration is shortened while seizure latency is prolonged in the mice infected with rAAV-GfaABC1D-sh.NFIA, compared with the mice infected with rAAV-GfaABC1D-sh.NC (Fig. 9B). EEG recordings showed that 4-AP-induced aberrant discharge is profoundly inhibited in the mice infected with rAAV-GfaABC1D-sh.NFIA (Fig. 9C). Likewise, HE and Nissl staining results also exhibited that 4-AP-induced neuronal damage is alleviated in DG and hilus regions of hippocampal tissues from the mice infected with rAAV-GfaABC1D-sh.NFIA (Fig. 9D, E).

## Discussion

**Fig. 10:**
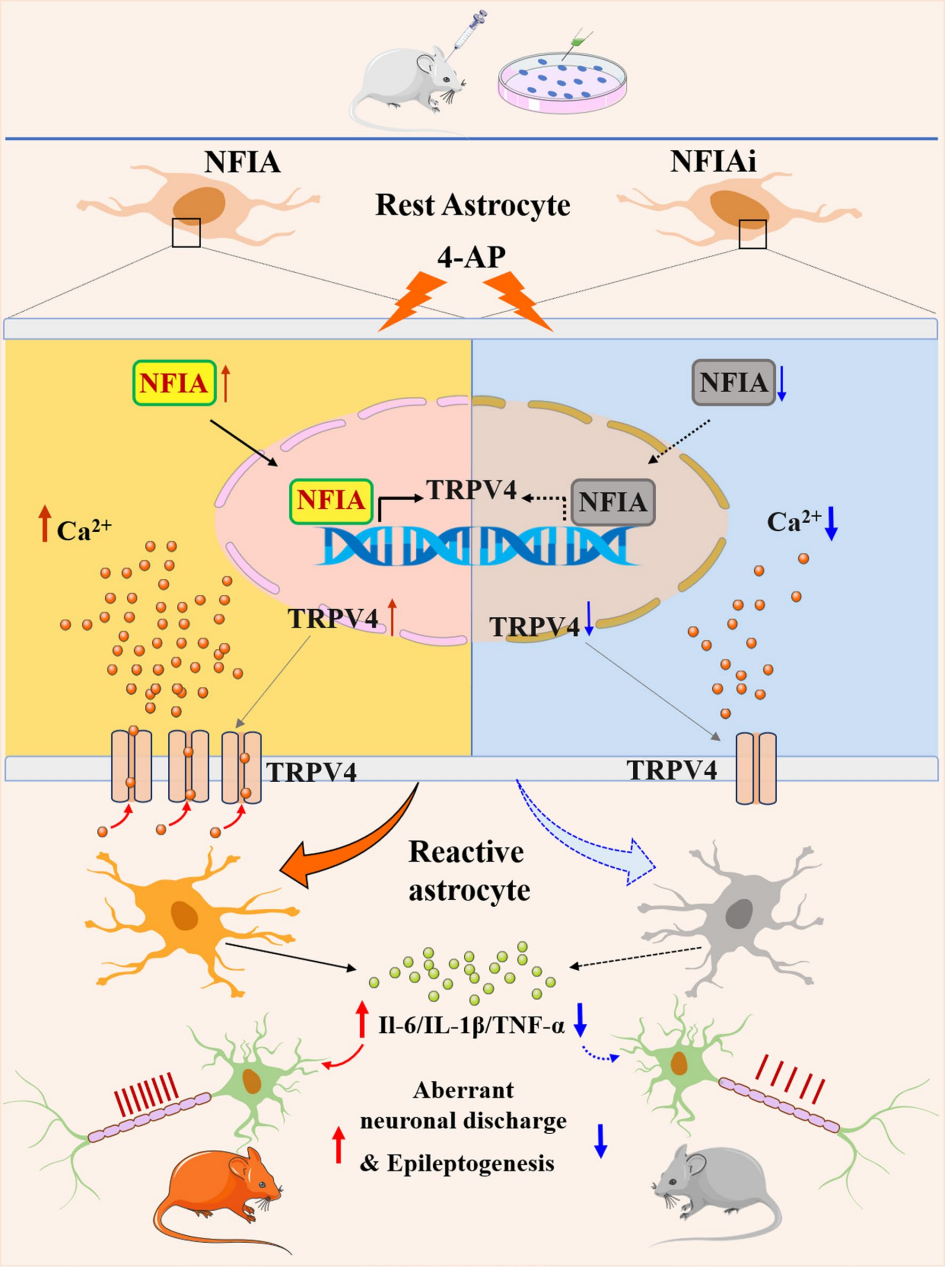
Schematic diagram of NFIA-TRPV4 signal mediating astrocyte reactivity to promote 4-AP induced epilepsy in mice. (left) In exposure of 4-AP, NFIA in astrocytes is upregulated and binds to TRPV4 promoter to increase TRPV4 expression, thereby remodeling astrocyte reactivity and increasing the expression of its proinflammatory cytokines, aggravating neuroinflammation to increase aberrant neuronal discharge, epileptic symptom and neuronal damage. (right) NFIA deficiency in astrocytes downregulates TRPV4 expression and inhibits the release of proinflammatory cytokines from reactive astrocyte, therefore attenuating neuroinflammation to improve epileptic symptoms and neuronal loss

In this study, we validated that the transcription factor NFIA in a cell-specific pattern promotes hippocampal astrocyte reactivity in a 4-AP-induced epileptic mouse model. Using NFIA expression silencing and overexpression approaches, we identified NFIA as a critical transcriptional regulator for 4-AP-induced astrocytic TRPV4 expression in hippocampal region. Furthermore, overexpression of NFIA in astrocytes augmented the expression of inflammatory cytokines via the functional activity of TRPV4, thus aggravating neuroinflammation and exacerbating epilepsy. Accordingly, the knockdown of NFIA meliorated 4-AP-induced epileptic symptoms and attenuated aberrant neuronal discharge and neuronal damage (Fig. 10). Our study shows that cell-specific NFIA upregulation drives astrocytic biological behavior to unbalanced neural circuit activity, facilitating epileptogenesis.

Clinically, the patients with hippocampal epileptogenic insult have a higher risk for recurrence after a first seizure due to neuronal damage and reactive astrogliosis in the hippocampus [3]. These hippocampal pathological processes are also observed in 4-AP-induced mouse model and become the trigger for seizure recurrent [18, 22–24]. A central question for blocking seizure recurrent is how epileptogenic insult maps genetic program to initiate corresponding signaling pathway. Previous study reported seizure was a very common symptom in NFIA haploinsufficiency patients [28]. Surprisingly, in this study, under the help of stereo-location, the in vivo si.NFIA delivery into mouse hippocampal region reduces neuronal damage and alleviates 4-AP-induced seizure grade, not completely blocks seizure (Fig. 1), which is convenient for us to explore the role of NFIA in seizure development. The electrophysiological recordings in brain slices showed that knocking down NFIA in hippocampal region can prevent pyramidal neurons from 4-AP-evoked overexcitation upon current stimulation (Fig. 2). The breakdown of neuronal excitation and inhibition balance may be caused by diverse factors derived from neuron itself or glia [29–32]. NFIA is widely expressed in neurons in hippocampal regions, especially in DG region rich in neuronal precursors (Additional file 1: Fig. S2), which is consistent with the previous description [17]. However, the increase of hippocampal NFIA^+^ cell number induced by 4-AP appears neither in neurons nor in microglia, instead in astrocytes (Fig. 3 and Additional file 1: Fig. S2). Our results implicate this cell specificity of NFIA upregulation may be a genetic strategy performed by the hippocampus after epileptogenic insult. To further validate the critical role of astrocytic NFIA in 4-AP-induced seizure model, rAAV targeting astrocytic NFIA was injected into hippocampal region. Similarly, 4-AP-induced seizure was alleviated in the mice infected with an astrocyte-specific rAAV-GfaABC1D-sh.NFIA, with decreased seizure grade and shortened seizure duration and prolonged seizure latency. Moreover, 4-AP-induced hippocampal neuronal damage could be also mitigated by astrocytic NFIA knockdown (Fig. 9 and Additional file 1: Fig. S7). These findings further substantiated that the contribution of NFIA upregulation to 4-AP-induced seizure in mice is astrocyte-specific.

It has been reported that NFIA plays region- and injury-specific roles in reactive astrocytes, alternatively promotes pro-inflammatory or anti-inflammatory action [17]. In this study, the astrocytic C3 was increased in a 4-AP-induced epileptic mouse model, and this augmentation of C3 could be curbed by NFIA deficiency caused by specific siRNA (Figs. 4, 5). In contrast, no apparent change was found in the expression of S100A10. These results suggested that NFIA defines reactive astrocyte subpopulation with pro-inflammatory action after 4-AP insult.

Although the role of NFIA in astrocyte reactivity is undeniable, it possesses distinct downstream target molecules and may initiate different transcriptional regulatory cascade to affect this reactivity [33, 34]. Furthermore, there is also much proof that calcium channels including multiple members of TRPV may initiate calcium signaling pathways to remodel astrocyte responses [35, 36]. In particular, the molecular remodeling of nonselective ion channels on the membrane of astrocytes may result in hyperexcitability of neurons and epileptogenesis [37]. TRPV4 in the CNS is expressed mainly in astrocytes, followed by microglia and neurons [38]. In this study, strikingly, NFIA and TRPV4 expression were upregulated coincidently in hippocampal astrocytes from TLE surgical patients and 4-AP-induced seizure mice (Fig. 5), and primary cultured astrocytes treated with 4-AP (Additional file 1: Fig. S4). NFIA knockdown profoundly inhibits 4-AP-induced TRPV4 expression and TRPV4-mediated calcium entry (Fig. 6) in primary cultured hippocampal astrocytes. Based on these observations and the phenomenon in our recent study that 4-AP-induced TRPV4 upregulation has similar cell-specific feature as NFIA [18], we speculate that NFIA may directly regulate TRPV4 expression. As expected, NFIA combined with the promoter region of the TRPV4 gene transcriptionally promoted its expression in astrocytes (Fig. 7).

Astrocytic TRPV4 activity can upregulate the expression of proinflammatory factors such as IL-6, IL-1β, and TNF-α to mediate neuroinflammation, thus leading to neuronal impairment and death [1, 39–42]. It is necessary to determine whether the functional activity of TRPV4 is required for NFIA-modulated astrocyte pro-inflammatory responses in 4-AP-induced seizure. Using a plasmid overexpressing NFIA and a specific TRPV4 antagonist, we found that TRPV4 antagonist almost completely abolished 4-AP-induced C3 upregulation, although its inhibitory effect on GFAP expression is not significant, which does not deny the importance of functional activity of TRPV4 in astrocyte reactivity. And this required function of TRPV4 is proved further by the weakened proinflammatory cytokines such as IL-1β, IL-6 and TNF-α from reactive astrocytes in the presence of TRPV4 antagonist (Fig. 8).

## Conclusions

Collectively, our results validate that NFIA transcriptionally regulate the expression of TRPV4 and disclose firstly that NFIA and TRPV4 coordinate to dictate hippocampal astrocyte responses after 4-AP insult in a cell-specific pattern, which may aid in our understanding how human epileptogenic insult maps genetic programs to aggravate epileptogenesis and provides a promising therapeutic strategy for the development of antiseizure drugs that can limit seizure recurrent.

### Supplementary Information

**Additional file 1:** **Figure S1.** The interference efficiency measurement of si.NFIA in mice. (**A**) Schema of si.NC/ NFIA delivery into the hippocampus region in mice. Representative immunoblot bands of NFIA (**Bi**) and its band density analysis (**Bii**) (*n =* 3, *** *P* < 0.001, One-way ANOVA). **Figure S2.** Effects of intraperitoneal 4-AP on NFIA expression in mouse hippocampal neurons and microglia. **A** Representative confocal images showing NFIA^+^ neurons and **B** NFIA^+^ microglia in hippocampal CA1, CA2, CA3 and DG from the mice with ip.4-AP or saline. (*n =* 6 for each group, Student’s *t* test). **Figure S3.** Effects of NFIA deficiency on the conversion of the astrocytic phenotype induced by 4-AP. **A** Scheme of primary astrocyte preparation for immunofluorescent staining and protein analysis. **B** Representative confocal images (**Bi**) of GFAP and C3 and the fluorescence intensity analysis of (**Bii**) GFAP and (**Biii**) C3 in primary cultured astrocytes transfected with si.NC or si.NFIA in the presence or absence of 4-AP (*n =* 6 for each group, ***p < 0.001, One-way ANOVA)*.* Representative immunoblot bands (**Ci**) of S100A10, GFAP, NFIA and C3, and the band density analysis of NFIA (**Cii**), GFAP (**Ciii**), S100A10 **(Civ)** and C3 (**Cv**) in primary cultured astrocytes transfected with si.NC or si.NFIA in 4-AP exposure or not (*n =* 3 for each group, **P < 0.01, ***P < 0.001, One-way ANOVA). **Figure S4.** Effects of 4-AP on the expression of NFIA and TRPV4 in mouse hippocampal tissues and in primary cultured astrocytes. **A** The qPCR analysis showing mRNA levels of NFIA (**Ai**) and TRPV4 (**Aii**) in the hippocampus from the mice with intraperitoneal 4-AP or saline injection (*n =* 5 for each group. * *P* < 0.05, ** *P* < 0.01, Student’s *t* test). Representative immunoblot bands (**Bi**) and the band intensity analysis exhibiting protein levels of GFAP (**Bii**), NFIA (**Biii**) and TRPV4 (**Biv**) in hippocampal tissues from the mice with intraperitoneal 4-AP or saline injection (*n =* 3 for each group, *** *P* < 0.001, Student’s *t* test). Representative confocal immunofluorescent images of NFIA (**Ci**) and TRPV4 (**Cii**) and their immunofluorescence intensity analysis in primary cultured astrocytes with or without 4-AP treatment (*n =* 6 for each group, ***P < 0.001, Student’s *t* test). The qPCR analysis of NFIA (**Di**) and TRPV4 (**Dii**) mRNA levels in primary cultured astrocytes with or without 4-AP treatment (*n* = 4 for each group, *P < 0.05, **P < 0.01, Student’s *t* test). Representative immunoblot bands (**Ei**) of NFIA and TRPV4 in primary cultural astrocytes with or without 4-AP treatment and the band density analysis of NFIA (**Eii**), GFAP (**Eiii**) and TRPV4 (**Eiv**) (*n* = 3 for each group, *P < 0.05, **P < 0.01, ***P < 0.001, Student’s *t* test). **Figure S5.** Effects of hippocampal si.NFIA treatment on the expression of TRPV4. **A** Representative immunoblot bands of NFIA and TRPV4 in hippocampal tissues from the mice treated with si.NC or si.NFIA and their band density analysis (**B**) (*n* = 3 for each group, ***P < 0.001, Student’s *t* test). **Figure S6.** Effects of astrocytic NFIA knockdown on TRPV4 upregulation and cytosolic calcium level. **A** Representative immunoblot bands of NFIA and TRPV4, and the band density analysis of NFIA and TRPV4 in astrocytes transfected with si.NC or si.NFIA in the presence or absence of 4-AP (*n* = 3 for each group, **P < 0.001, ***P < 0.001, ****P < 0.0001, One-way ANOVA). **Bi** Representative confocal images of the Ca^2+^ indicator Fluo 4 and the fluorescence intensity analysis (**Bii**) showing the basal Ca^2+^ level in primary cultured astrocytes transfected with si.NC or si.NFIA in the presence or absence of 4-AP (*n* = 8 for each group, *p < 0.05, ****p < 0.0001, One-way ANOVA). **Figure S7.** Hippocampal astrocytic NFIA knockdown via rAAV infection targeting astrocyte. **A** Scheme of experiments on hippocampal rAAV delivery and immunofluorescent staining. **B** Representative confocal images of immunofluorescent staining showing NFIA expression in rAAV-GfaABC1D-sh.NC-infected group versus rAAV-GfaABC1D-sh. NFIA-infected group.

## Data Availability

All data used in this manuscript are available from the corresponding author on reasonable request.

## Abbreviations

CNS: Central nervous system
TLE: Temporal lobe epilepsy
NFIA: Nuclear factor I-A
TRPV4: Vanilloid transient receptor potential 4
GFAP: Glial fibrillary acidic protein
4-AP: 4-Aminopyridine
EEG: Electroencephalogram
si.NFIA: Specific siRNA targeting NFIA
si.NC: Non-specific siRNA
AP: Action potential
HE: Hematoxylin–eosin
HRP: Horseradish peroxidase
FBS: Fetal bovine serum
HA-NFIA: HA-tagged plasmid overexpressing NFIA
BSA: Bovine serum albumin
TBST: Tris-buffered saline containing 0.2% Tween-20
qPCR: Quantitative real-time polymerase chain reaction
ChIP: Chromatin immunoprecipitation
C3: The third component of complement
TNF-α: Tumor necrosis factor-α
IL-6: Interleukin-6
IL-1β: Interleukin-1 beta
