# Supplementary figures and images for: Cell-specific NFIA upregulation promotes epileptogenesis by TRPV4-mediated astrocyte reactivity
Source: J Neuroinflammation. 2023 Oct 25;20:247. doi: 10.1186/s12974-023-02909-4 (PMC10601220; doi:10.1186/s12974-023-02909-4)

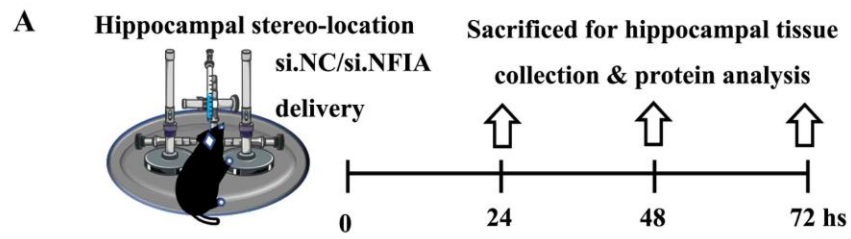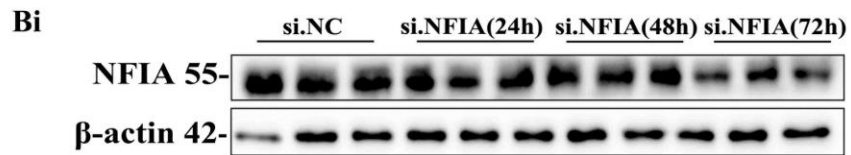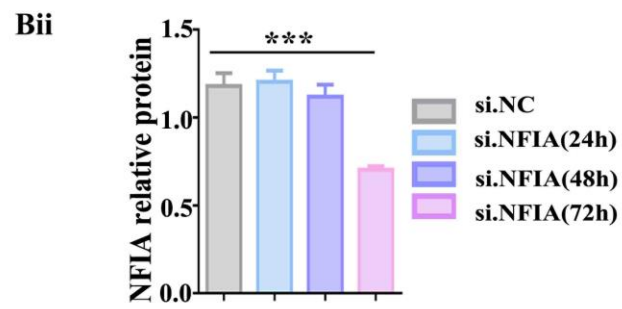

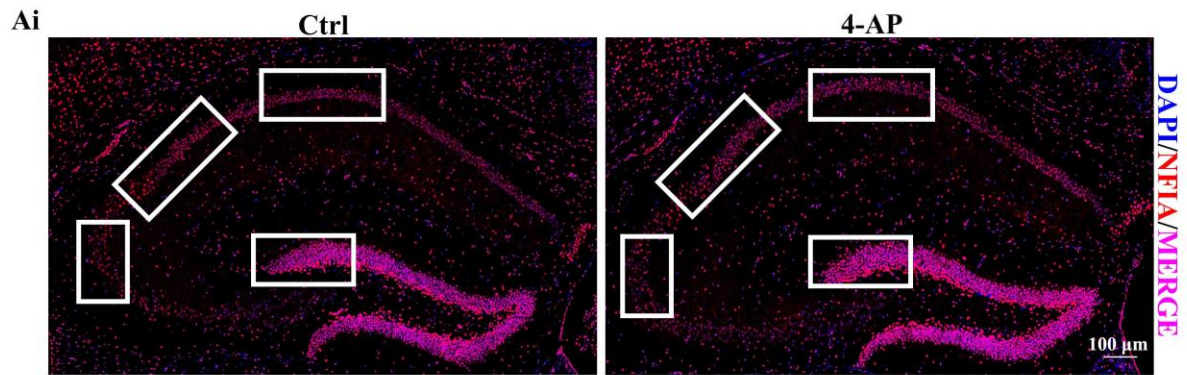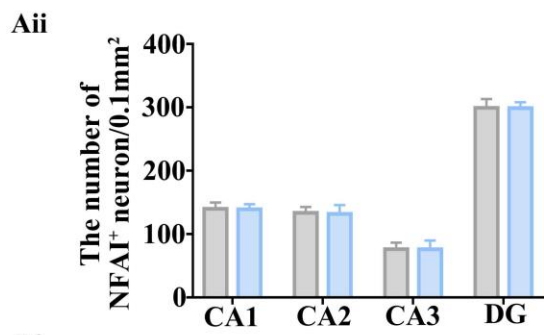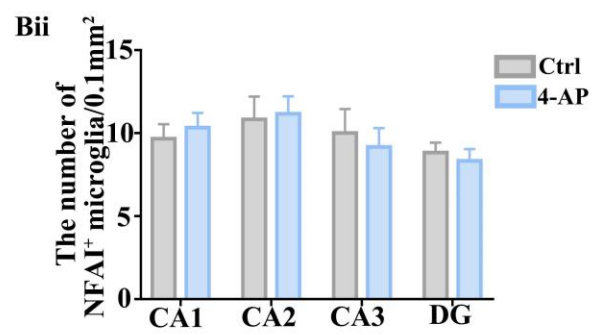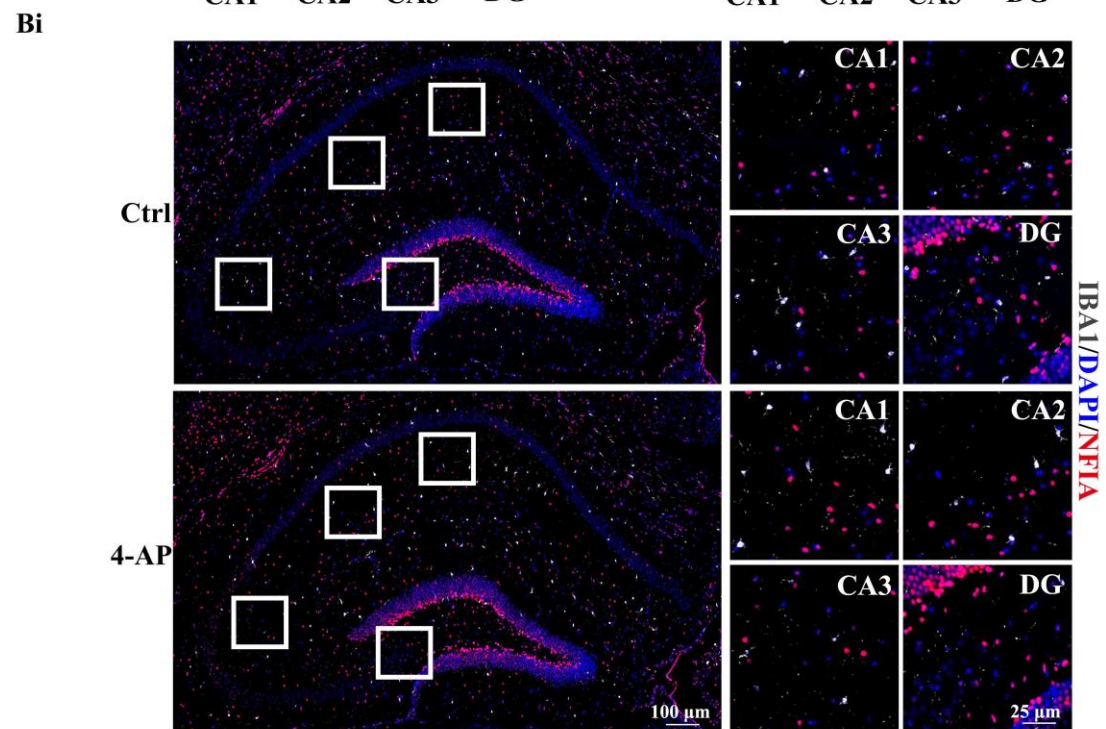

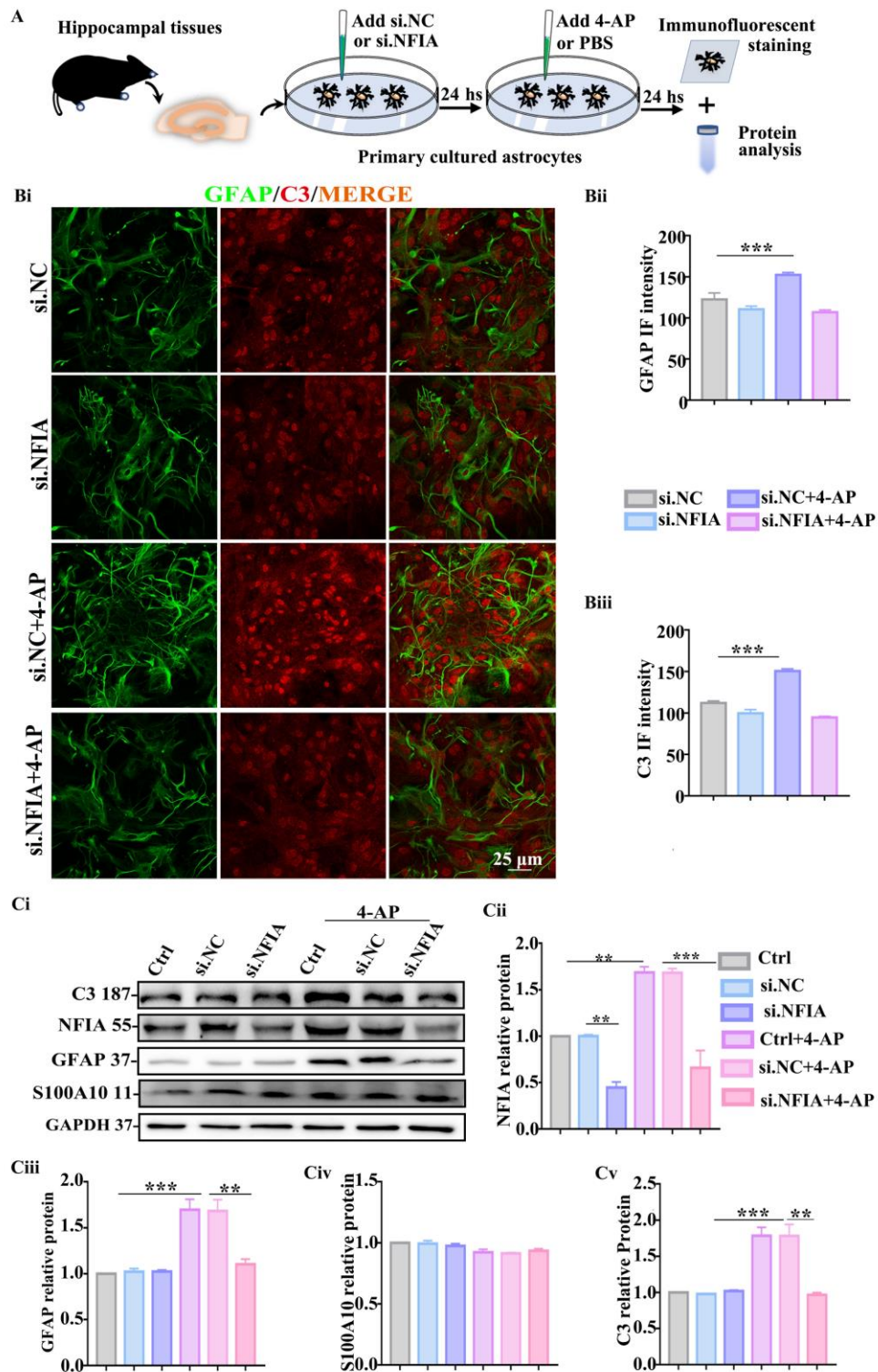

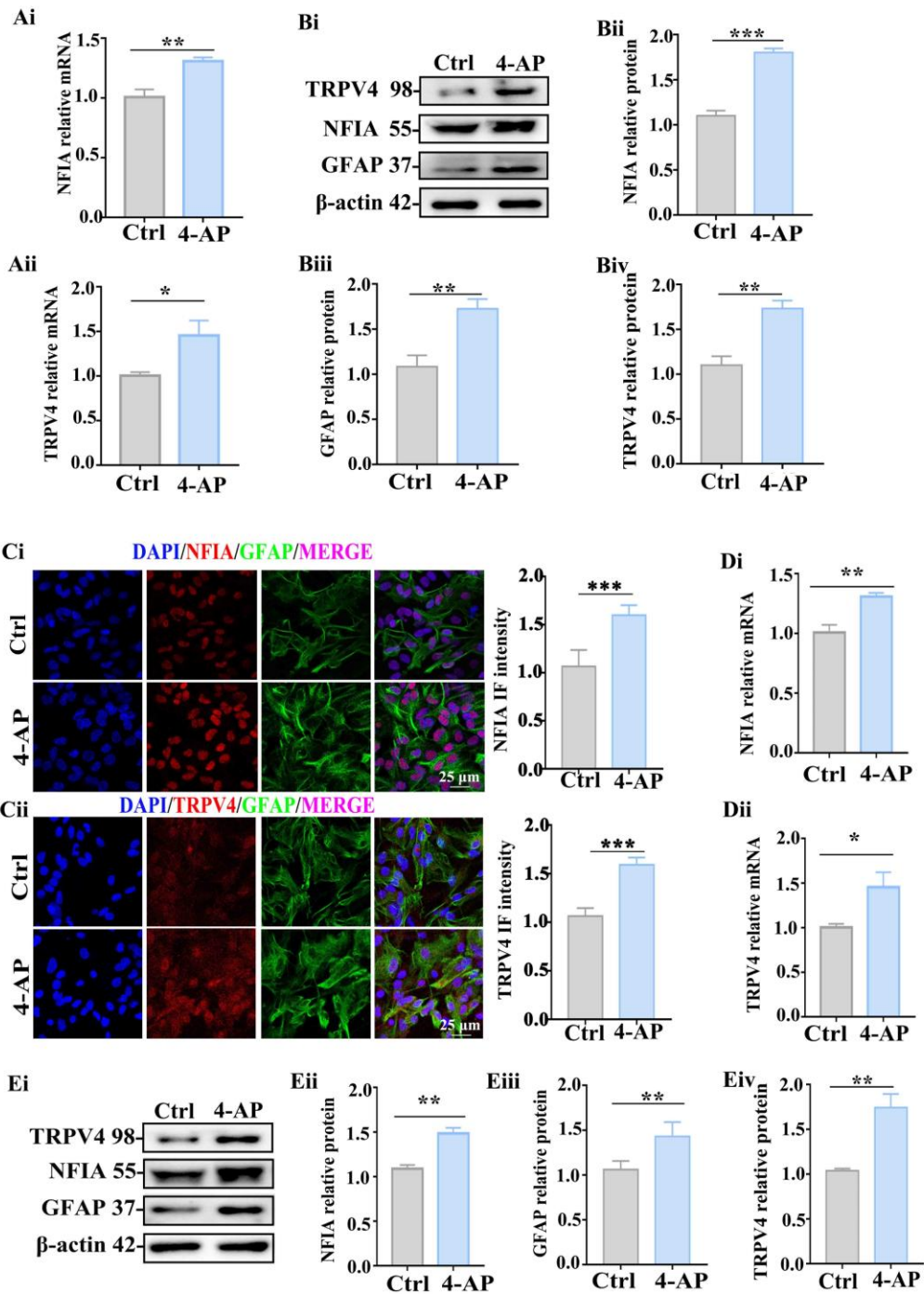

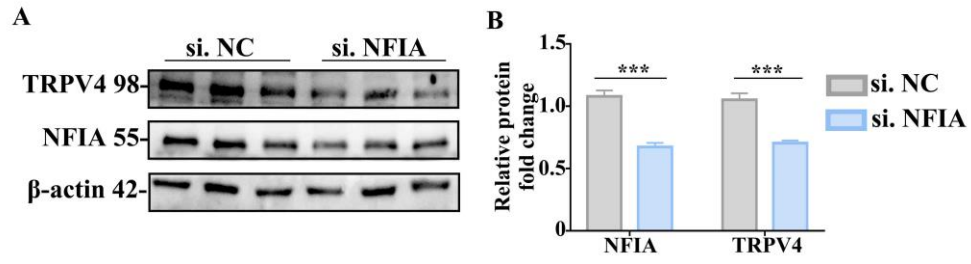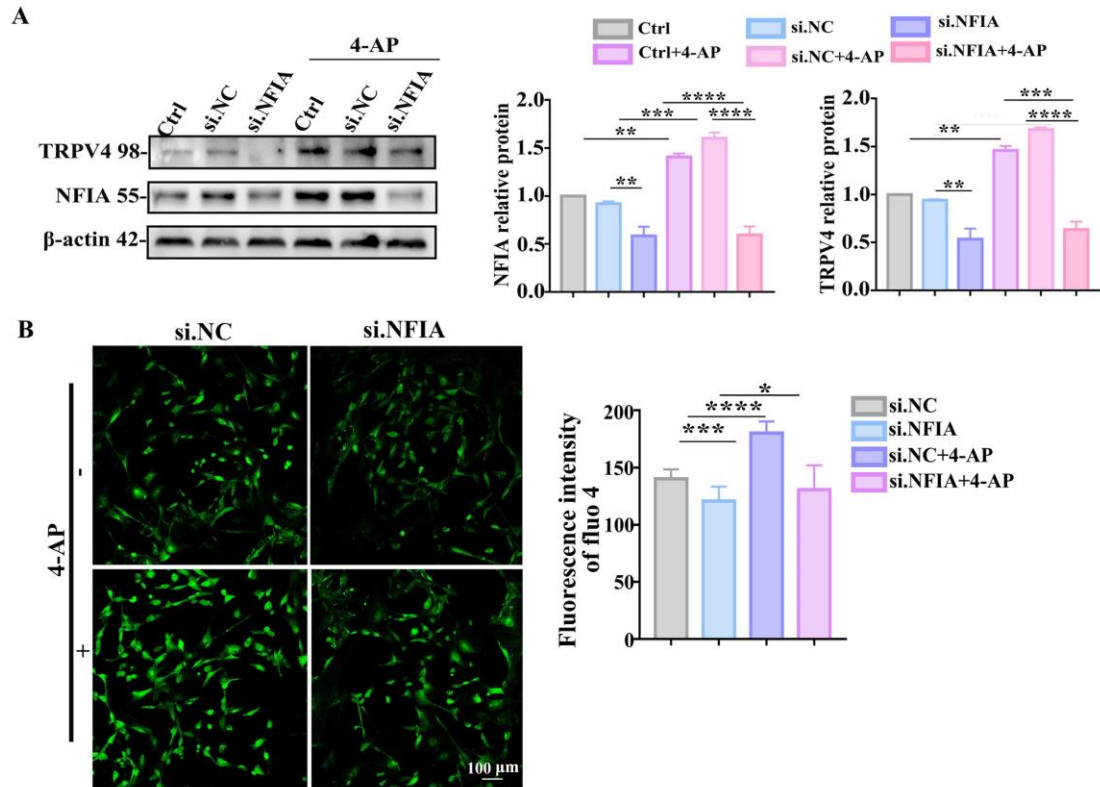

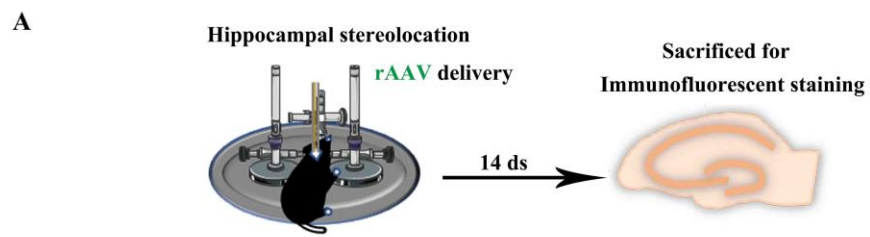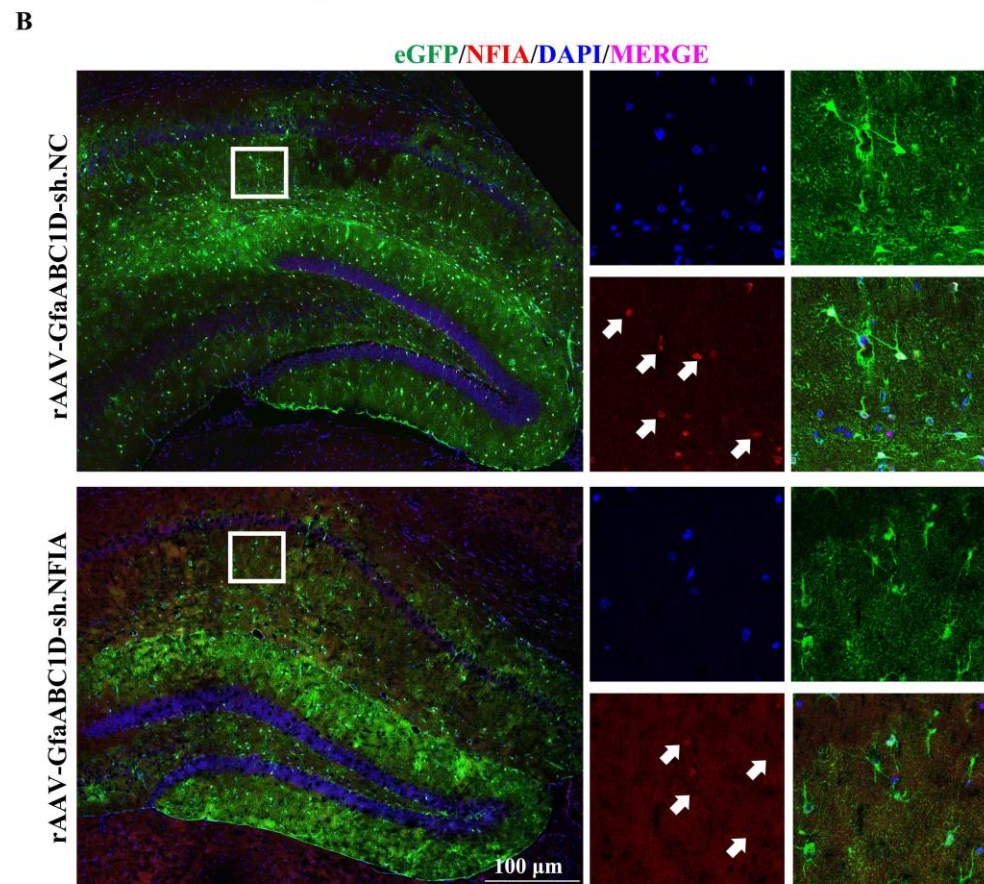

Supplement: Supplementary file 1 — Additional file 1: Figure S1. The interference efficiency measurement of si.NFIA in mice. (A) Schema of si.NC/ NFIA delivery into the hippocampus region in mice. Representative immunoblot bands of NFIA (Bi) and its band density analysis (Bii) (n = 3, *** P < 0.001, One-way ANOVA). Figure S2. Effects of intraperitoneal 4-AP on NFIA expression in mouse hippocampal neurons and microglia. A Representative confocal images showing NFIA+ neurons and B NFIA+ microglia in hippocampal CA1, CA2, CA3 and DG from the mice with ip.4-AP or saline. (n = 6 for each group, Student’s t test). Figure S3. Effects of NFIA deficiency on the conversion of the astrocytic phenotype induced by 4-AP. A Scheme of primary astrocyte preparation for immunofluorescent staining and protein analysis. B Representative confocal images (Bi) of GFAP and C3 and the fluorescence intensity analysis of (Bii) GFAP and (Biii) C3 in primary cultured astrocytes transfected with si.NC or si.NFIA in the presence or absence of 4-AP (n = 6 for each group, ***p < 0.001, One-way ANOVA). Representative immunoblot bands (Ci) of S100A10, GFAP, NFIA and C3, and the band density analysis of NFIA (Cii), GFAP (Ciii), S100A10 (Civ) and C3 (Cv) in primary cultured astrocytes transfected with si.NC or si.NFIA in 4-AP exposure or not (n = 3 for each group, **P < 0.01, ***P < 0.001, One-way ANOVA). Figure S4. Effects of 4-AP on the expression of NFIA and TRPV4 in mouse hippocampal tissues and in primary cultured astrocytes. A The qPCR analysis showing mRNA levels of NFIA (Ai) and TRPV4 (Aii) in the hippocampus from the mice with intraperitoneal 4-AP or saline injection (n = 5 for each group. * P < 0.05, ** P < 0.01, Student’s t test). Representative immunoblot bands (Bi) and the band intensity analysis exhibiting protein levels of GFAP (Bii), NFIA (Biii) and TRPV4 (Biv) in hippocampal tissues from the mice with intraperitoneal 4-AP or saline injection (n = 3 for each group, *** P < 0.001, Student’s t test). Representative confoca [file 12974_2023_2909_MOESM1_ESM.pdf]
